# Lactate Drives Pathological Browning of White Adipose Tissue to Aggravate Sepsis‐Associated Metabolic Disorders in a GPR81‐Dependent Manner

**DOI:** 10.1002/advs.78487

**Published:** 2026-10-29

**Authors:** Chunliang Zheng, Xiaohua Li, Shuo Li, Chungen Xing, Chun Cao

**Affiliations:** ^1^ Department of General Surgery the Second Affiliated Hospital of Soochow University Suzhou China; ^2^ Department of Thyroid and Breast Surgery Suzhou Wuzhong People's Hospital Suzhou China

**Keywords:** GPR81‐CTRP6‐p38 pathway, lactate, metabolic disorders, sepsis, UCP1, white adipose tissue browning

## Abstract

Lactate serves as a prognostic biomarker for sepsis, yet its adipose‐centered signaling pathway driving persistent metabolic disorders remains unclear. We reveal that lactate accumulation triggers browning of white adipose tissue (WAT) via GPR81 to aggravate sepsis‐induced hypermetabolism, muscle wasting, and insulin resistance. Septic patients exhibit hyperlactatemia and WAT browning, with lactate concentrations correlated with fat and muscle loss. Genetically deleting UCP1 substantially attenuates LPS‐induced hypermetabolism, muscle wasting, and insulin resistance, confirming WAT browning as a major mediator of sepsis‐associated metabolic disorders. Lactate supplementation and pharmacological interventions using glycolysis or lactate dehydrogenase inhibitors identify lactate as the upstream pathogenic trigger. Using siRNA screening, global GPR81 knockout, and AAV9‐mediated adipose‐specific GPR81 knockdown, we identify GPR81 as the lactate receptor mediating UCP1 upregulation. Mechanistically, GPR81 activation induces CTRP6 and p‐p38 to upregulate UCP1. AAV9‐mediated overexpression of CTRP6/MKK6 in GPR81^−/−^ mice, together with pharmacological downregulation of CTRP6 or inhibition of p‐p38, supports the involvement of this pathway in WAT browning. Taken together, evidence from clinical data, gene‐knockout mice, and primary adipocyte experiments demonstrates that lactate drives WAT browning in a GPR81‐dependent manner to aggravate sepsis‐associated metabolic disorders, identifying potential targets for therapeutic investigation in metabolic disorders unresponsive to conventional anti‐infective and nutritional support treatments.

## Graphical abstract

Sepsis‐triggered lactate accumulation induces pathological white adipose tissue browning. Lactate activates adipocyte GPR81 to sequentially upregulate CTRP6‐p38 signalling and UCP1 expression. This cascade exacerbates sepsis‐associated metabolic disorders.

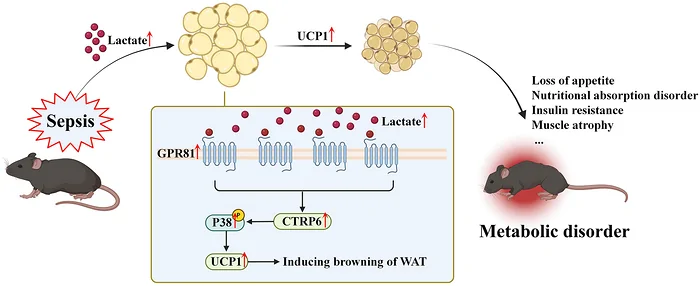

## 1 Introduction

As a major global public health burden, sepsis remains one of the leading causes of mortality worldwide [1]. Sepsis is characterized by life‐threatening systemic inflammatory responses and profound immune dysfunction triggered by host infection [2]. A robust pro‐inflammatory cytokine storm during sepsis drives a systemic hypermetabolic state [3]. Septic patients experience anorexia, skeletal muscle protein breakdown and insulin resistance in a short period of time, ultimately leading to dysfunction of multiple organs such as the heart, lungs, and kidneys [4]. Dysregulated lipid, glucose, and protein metabolism act synergistically to disrupt whole‐body energy homeostasis, exacerbating tissue hypoxia and inflammatory damage and creating a vicious cycle that accelerates multi‐organ failure [5]. Even in sepsis survivors, long‐lasting metabolic disorders including glucose intolerance, fat redistribution, and lean body mass loss commonly persist for months or years post‐discharge, driving chronic post‐sepsis sequelae and imposing substantial healthcare burdens [1, 3]. Standard clinical interventions, including anti‐infective treatment, organ support, and nutritional supplementation, can effectively control primary infection and suppress exaggerated inflammatory responses yet fail to reverse established pathological metabolic disorders [6]. Meanwhile, the EPaNIC and EDEN studies have revealed that conventional enteral and parenteral nutrition cannot effectively attenuate metabolic disorders in septic patients; conversely, excessive nutritional supplementation may increase the risks of complications and mortality [7, 8]. Thus, elucidating the molecular mechanisms underlying sepsis‐associated metabolic disorders during the stress period is critical for improving therapeutic management and clinical prognosis in septic patients.

Notably, adipose tissue acts as a central hub linking energy metabolism and immune regulation, and mediates sepsis‐induced systemic inflammation and metabolic dysfunction [9]. Distinct adipose tissue subtypes exert unique functions in energy storage and metabolic homeostasis. White adipose tissue (WAT) primarily functions as an energy depot to sustain metabolic homeostasis, while brown adipose tissue generates heat efficiently and adapts to shifts in systemic internal and external environmental cues [10]. Clinical studies have demonstrated that subcutaneous and visceral WAT undergoes characteristic browning transformation in septic patients, marked by smaller multilocular adipocytes, upregulated uncoupling protein 1 (UCP1) expression, increased mitochondrial density, and augmented respiratory capacity [11]. Consistent with findings from cecal ligation and puncture (CLP)‐induced sepsis mouse models, acute inflammatory responses are robustly activated in adipose tissue during sepsis, accompanied by pronounced transcriptional upregulation of thermogenic genes including UCP1 [12]. Clinical studies have revealed that critically ill patients exhibit concurrent WAT browning accompanied by persistent energy overconsumption and progressive weight loss [13]. Taken together, these observations indicate that WAT browning may be an important mechanism responsible for sepsis‐associated metabolic disorders.

Clinically, lactate is widely recognized as a key biomarker of tissue hypoxia and persistently elevated lactate concentrations correlate positively with organ failure and mortality in septic patients [14, 15]. However, some critically ill patients present hyperlactatemia without apparent tissue hypoperfusion, suggesting that overproduction of lactate is not simply caused by tissue hypoxia [16, 17]. Sepsis‐induced glucocorticoid resistance and impaired hepatic gluconeogenesis prevent efficient hepatic lactate clearance, leading to systemic lactate accumulation, as lactate represents the primary byproduct of aerobic glycolysis [18]. Accumulated lactate can promote lactylation and exosomal release of high mobility group box‐1 in macrophages, thereby increasing sepsis‐induced vascular permeability [19]. Furthermore, the high concentrations of lactate in septic patients suppress immune function, raise secondary infection risk, and further exacerbate organ dysfunction [20]. Lactate significantly upregulates functional UCP1 to drive WAT browning and lipolysis in models of burn injury and cancer cachexia [13, 21]. Collectively, lactate concentrations and their dynamic fluctuations correlate tightly with disease severity and multi‐organ dysfunction risk, defining lactate as a vital biomarker for monitoring disease progression and guiding therapeutic strategies during sepsis [22]. G protein‐coupled receptor 81 (GPR81) is highly expressed in adipose tissue and acts as the primary lactate receptor governing adipocyte metabolic homeostasis [23]. Suppressing endogenous lactate production or blocking GPR81‐mediated signaling pathways in macrophages improves the survival rate of septic mice19. Our prior work demonstrated that octanoic acid‐rich enteral nutrition (OA‐rich EN) attenuates sepsis‐associated metabolic disorders by suppressing endogenous lactate production and downregulating GPR81 expression in WAT, which blunts the downstream activation of C1q/tumor necrosis factor‐related protein 6 (CTRP6) and subsequent p38 phosphorylation [24]. p38 phosphorylation is required for UCP1 transcriptional activation, as it promotes activating transcription factor 2 nuclear translocation and subsequent robust upregulation of UCP1 protein [25]. Although our prior work demonstrated that an OA‐rich EN intervention that lowered lactate and GPR81 also reduced CTRP6 and p‐p38 in septic mice, that study was observational in nature [24]. It could not establish whether lactate and GPR81 are causally required, which cell type mediates this effect, the sequential order of the downstream signaling cascade, or whether this pathway operates in human subjects. Furthermore, although lactate‐driven WAT browning has been documented in burn injury and cancer cachexia [13, 21], receptor‐effector coupling for this pathway has not been validated using genetic tools under septic conditions.

Here, we delineate the core molecular mechanism through which lactate aggravates sepsis‐associated metabolic disorders by driving pathological WAT browning in a GPR81‐dependent manner. This adipocyte‐intrinsic regulatory process is mediated via the lactate‐GPR81‐CTRP6‐p38 pathway. Multi‐dimensional evidence from clinical data, gene‐knockout mouse models and primary white adipocyte experiments fills the knowledge gap linking sepsis‐induced lactate accumulation to metabolic disorders and identifies potential targets for future therapeutic investigation in metabolic disorders unresponsive to conventional anti‐infective and nutritional support treatments.

## 2 Result

### 2.1 Lactate Accumulation and Pathological WAT Browning are Closely Correlated With Metabolic Disorders in Septic Patients

Lactate served as a key biomarker for severe sepsis and septic shock, and its concentrations correlated tightly with disease severity and patient prognosis [26]. We enrolled cholelithiasis patients with or without sepsis to characterize clinical correlations among lactate accumulation, pathological WAT browning, and sepsis‐associated metabolic disorders. Septic patients had significantly higher SOFA and APACHE II scores, increased inflammatory marker levels, abnormal liver function, abnormal renal function, and hypoalbuminemia (Table S1). On postoperative day 3, quantitative abdominal computed tomography (CT) at the third lumbar (L3) vertebral level demonstrated markedly larger reductions in total abdominal muscle tissue (TAMT) (effect estimates, ‐5.79; 95% confidence interval, −11.42–−0.17; p = 0.0443), total adipose tissue (TAT) (effect estimates, −13.26; 95% confidence interval, ‐23.43 to ‐3.10; p = 0.0134), subcutaneous adipose tissue (SAT) (effect estimates, −12.63; 95% confidence interval, −24.61–−0.66; p = 0.0496), and visceral adipose tissue (VAT) (effect estimates, −10.62; 95% confidence interval, −21.00–−0.25; p = 0.0453) in septic versus non‐septic patients (Figure 1A–E). These P values are close to the conventional threshold and should be interpreted cautiously. Serum lactate concentration (effect estimates, 7.95; 95% confidence interval, 6.10 to 9.80; p < 0.001) and omental WAT lactate concentration (effect estimates, 0.62; 95% confidence interval, 0.51 to 1.22; p < 0.001) were substantially higher in septic patients relative to non‐septic counterparts (Figure 1F,G). Linear regression correlation analysis identified strong negative associations between lactate concentrations in serum and WAT and total adipose tissue (TAT) loss, as well as weak negative correlations between lactate concentrations and TAMT loss (Figure 1H–K). Higher lactate concentrations corresponded to more profound fat and lean mass depletion in septic patients. Targeted UHPLC‐MRM‐MS energy metabolomics profiling of omental WAT detected robust upregulation of glycolysis and lactate metabolic pathways in septic patients (Figure 1L–N).

**FIGURE 1:**
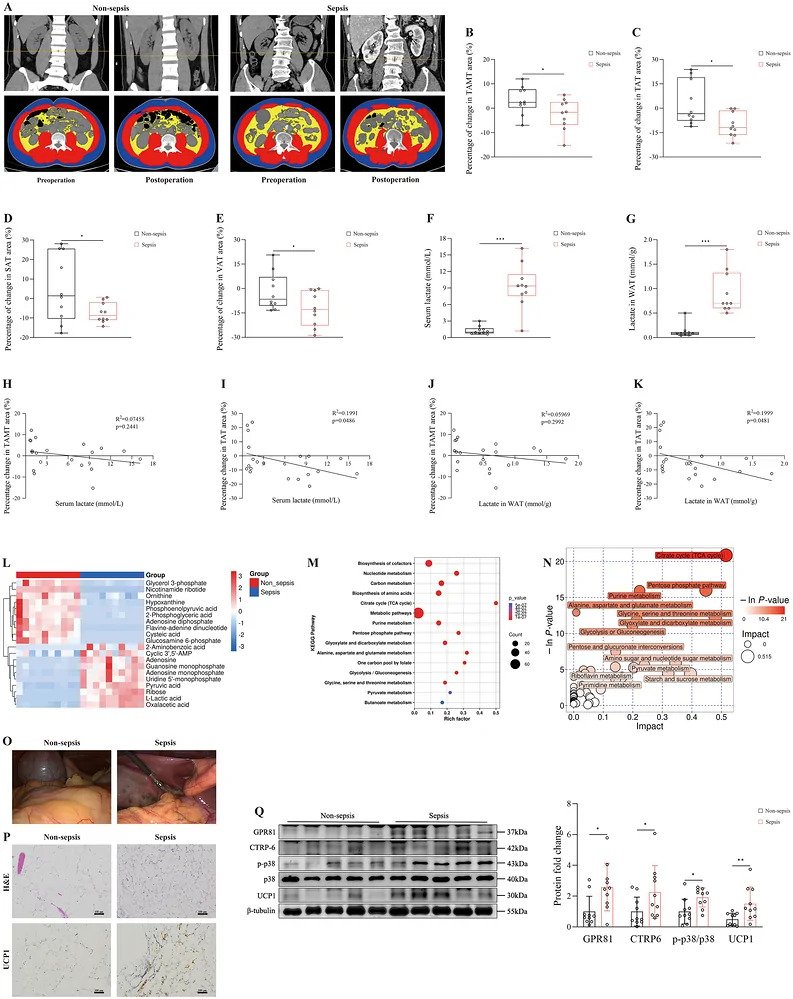
Association of lactate accumulation and pathological WAT browning with sepsis‐related metabolic disorders: clinical evidence. (A) Representative L3‐level abdominal CT cross‐sectional images from non‐septic and septic patients obtained preoperatively and on postoperative day 3 (*n* = 10 per group). Upper panels: coronal plane view of the L3 vertebral midsegment labeled with a yellow line; lower panels: cross‐sectional images at L3 with TAMT (red), SAT (blue), and VAT (yellow). (B–E) Quantitative analysis of percentage changes in TAMT, TAT, SAT, and VAT on postoperative day 3 in septic versus non‐septic patients. (F,G) Lactate concentrations in serum and omental WAT. (H–K) Linear regression analyses evaluating associations between lactate concentrations in serum and omental WAT and cross‐sectional area changes in TAMT and TAT. (L) Heatmap generated from targeted energy metabolomic profiling of omental WAT. (M,N) KEGG enrichment bubble plots illustrating altered metabolic pathways in omental WAT. (O) Representative images of intraoperative omental WAT. (P) Representative images of H&E staining and UCP1 immunohistochemistry in omental WAT sections. (Q) Western blot analysis of GPR81, CTRP6, p‐p38, and UCP1 protein expression in omental WAT. Data are expressed as mean ± SEM. ^*^ *p* < 0.05, ^**^ *p* < 0.01, ^***^ *p* < 0.001. L3, the third lumbar vertebral; CT, computed tomography; TAMT, total abdominal muscle tissue; TAT, total adipose tissue; SAT, subcutaneous adipose tissue; VAT, visceral adipose tissue; WAT, white adipose tissue; H&E, Hematoxylin‐eosin; UCP1, uncoupling protein 1; GPR81, G protein‐coupled receptor 81; CTRP6, C1q/tumor necrosis factor‐related protein 6.

Intraoperative observations showed omental WAT from septic patients displayed a distinct reddish‐brown phenotype, whereas WAT from non‐septic patients appeared pale yellow (Figure 1O). Hematoxylin‐eosin (H&E) sections of WAT uncovered hallmark browning features in septic patients: markedly shrunken adipocytes packed with abundant small multilocular lipid droplets, in contrast to the large unilocular lipid droplets seen in non‐septic patients. Western blot analysis and immunohistochemistry confirmed that UCP1 expression was significantly upregulated in WAT from septic patients (Figure 1P,Q). Collectively, these clinical data demonstrated concurrent hyperlactatemia and pathological WAT browning among septic patients, with both phenotypes tightly correlated with systemic metabolic disorders.

### 2.2 Selection of the Lipopolysaccharide (LPS)‐Induced Sepsis Model Based on the Superior Stability of Metabolic Disorders Across Four Mouse Sepsis Models

To select a sepsis model with minimal inter‐mouse variability for subsequent metabolic studies, we compared four representative sepsis model: intraperitoneal injection of LPS, CLP, intraperitoneal injection of *Staphylococcus aureus* (*S. aureus*), and intranasal instillation of *S. aureus*. At 72 h post modeling, all four sepsis models exhibited elevated serum and WAT lactate concentrations, inflammatory responses, pathological WAT browning, and metabolic disorders (Figure S1). However, the LPS group showed consistent phenotypes across lactate and metabolic indicators, including serum and WAT lactate concentrations, total daily energy expenditure (EE), grip strength, and homeostatic model assessment of insulin resistance (HOMA‐IR). In contrast, the CLP and *S. aureus*‐infection groups exhibited marked inter‐individual heterogeneity in these indicators, with correspondingly higher coefficients of variation (CV) (Table S2). Although the CLP and *S. aureus*‐infection models better mimic polymicrobial sepsis, their high intrinsic variability would confound the interpretation of adipose‐centric metabolic phenotypes. Therefore, given its superior reproducibility of metabolic indicators, the LPS‐induced sepsis model was selected for all subsequent mechanistic investigations.

### 2.3 UCP1‐Mediated Pathological WAT Browning is a Major Driver of Sepsis‐Associated Metabolic Disorders

UCP1 knockout (UCP1^−/−^) mice were used to test whether UCP1‐mediated pathological WAT browning served as a key downstream mediator of sepsis‐induced metabolic disorders. Western blot analysis and immunohistochemistry showed undetectable UCP1 protein in WAT from UCP1^−/−^ mice with or without LPS administration, whereas significant UCP1 expression was observed in the wild‐type (WT) septic mice (Figure 2A,D). WAT histological and morphological assessments detected severe adipose atrophy and multilocular lipid droplet formation in the WT septic mice, whereas these pathological phenotypes characteristic of WAT browning were significantly reversed in UCP1^−/−^ mice (Figure 2B–E). 24‐h continuous metabolic cage monitoring identified severe negative energy balance in the WT septic mice, including reduced daily food consumption, body weight loss, and increased total daily EE, all LPS‐triggered energy disturbances were substantially attenuated in UCP1^−/−^ mice (Figure 2F–I). H&E sections of extensor digitorum longus (EDL) displayed severe skeletal muscle atrophy in the WT + LPS group, while myofiber structural integrity was preserved in the UCP1^−/−^ + LPS group (Figure 2J). Protein expression of the muscle atrophy markers, muscle ring‐finger‐1 (MURF‐1) and muscle atrophy F‐Box (MAFBx), was markedly increased in the WT + LPS group, and this LPS‐mediated upregulation was reversed in UCP1^−/−^ + LPS group (Figure 2K). Forelimb grip strength functional testing demonstrated significant skeletal muscle weakness in WT septic mice, which was attenuated in UCP1^−/−^ septic mice (Figure 2L). Fasting blood glucose, fasting serum insulin, and derived HOMA‐IR showed pronounced hypoglycemia, hyperinsulinemia, and robust insulin resistance in the WT septic mice; both hypoglycemia and insulin resistance were substantially attenuated in UCP1^−/−^ septic mice (Figure 2M–O). Knockout of UCP1 also attenuated the levels of IL‐1β, IL‐6, and TNF‐α in WAT and serum following LPS administration (Figure 2P,Q). Collectively, these data demonstrated that UCP1‐mediated pathological WAT browning constituted a major mediator of sepsis‐associated metabolic disorders.

**FIGURE 2:**
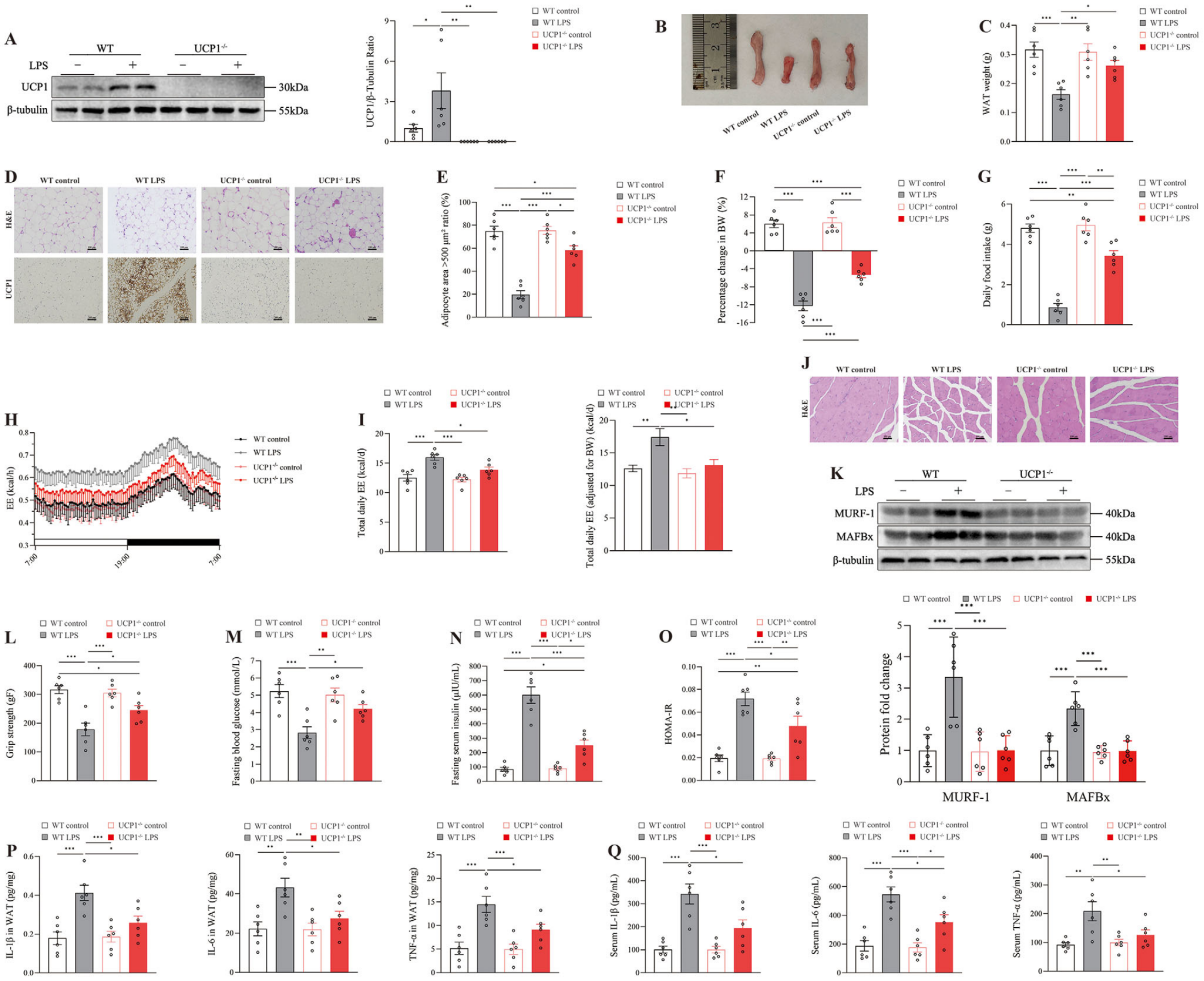
Genetically deleting UCP1 substantially attenuated LPS‐induced pathological WAT browning and systemic metabolic disorders in mice. (A) Western blot analysis of UCP1 in WAT from WT and UCP1^−/−^ mice subjected to intraperitoneal injection of saline or LPS (5 mg/kg) (*n* = 6 per group). (B) Representative images of WAT isolated from the inguinal region. (C) WAT weight. (D) Representative images of H&E staining and UCP1 immunohistochemistry in WAT sections. (E) Quantification of the proportion of adipocytes with a cross‐sectional area > 500 µm^2^. (F–I) Metabolic parameters continuously recorded by metabolic cages: percentage change in BW, daily food intake, 24‐h EE curve, and total daily EE (unadjusted data and data adjusted for body weight by ANCOVA). (J) Representative images of H&E staining in EDL sections. (K) Western blot analysis of MURF‐1 and MAFBx in EDL. (L) Forelimb grip strength measurement. (M–O) Fasting blood glucose, fasting serum insulin, and calculated HOMA‐IR for insulin resistance assessment. (P,Q) The levels of IL‐1β, IL‐6, and TNF‐α in WAT and serum were measured via ELISA. Data are expressed as mean ± SEM. ^*^ *p* < 0.05, ^**^ *p* < 0.01, ^***^ *p* < 0.001. WT, wild‐type; UCP1, uncoupling protein 1; WAT, white adipose tissue; LPS, lipopolysaccharide; H&E, Hematoxylin‐eosin; BW, body weight; EE, energy expenditure; EDL, extensor digitorum longus; MURF‐1, muscle ring‐finger‐1; MAFBx, muscle atrophy F‐Box; HOMA‐IR, homeostatic model assessment of insulin resistance; IL‐1β, interleukin‐1 beta; IL‐6, interleukin‐6; TNF‐α, tumor necrosis factor‐alpha.

### 2.4 Lactate Aggravates Sepsis‐Associated Metabolic Disorders via Driving WAT Browning

To establish lactate as the upstream causal driver of sepsis‐induced pathological WAT browning and subsequent metabolic disorders, we implemented two independent experimental strategies: exogenous lactate supplementation to raise systemic lactate concentrations in WT mice, and pharmacological inhibition of glycolysis or lactate dehydrogenase to suppress endogenous lactate production in both WT mice and stromal vascular fraction (SVF)‐derived adipocytes.

Co‐administration of LPS and lactate further increased lactate concentrations in serum and WAT (Figure 3A–B). Histological and morphological analyses of WAT showed that the LPS + Lactate group exhibited severe adipose atrophy and abundant multilocular lipid droplet formation compared with the LPS group (Figure 3C,D,H,I). Western blot analysis and immunohistochemistry showed that UCP1 expression was significantly increased in the LPS + Lactate group compared with the LPS group (Figure 3G,H). Exogenous lactate supplementation synergistically exacerbated LPS‐induced body weight loss, reduced food intake, and increased EE (Figure 3K,N–P). Linear regression correlation analysis identified strong positive associations between lactate concentrations in serum and WAT and total daily EE, and alongside strong negative associations between WAT weight and percentage change in body weight (Figure 3E,F,L,M,W–Y). Kaplan‐Meier survival analysis demonstrated that co‐administration of LPS and lactate significantly reduced 72‐h overall survival in septic mice (Figure 3J). EDL H&E staining showed that exogenous lactate supplementation worsened LPS‐induced muscular structural injury (Figure 3Q). Western blot analysis showed that co‐administration with LPS and lactate further upregulated LPS‐induced MURF‐1 and MAFBx expression (Figure 3R). Forelimb grip strength testing showed that LPS induced significant skeletal muscle weakness, a phenotype exacerbated by lactate co‐administration (Figure 3S). Exogenous lactate supplementation further disrupted glucose homeostasis and exacerbated insulin resistance and inflammatory response in septic mice (Figure 3T–V, Z‐AA).

**FIGURE 3:**
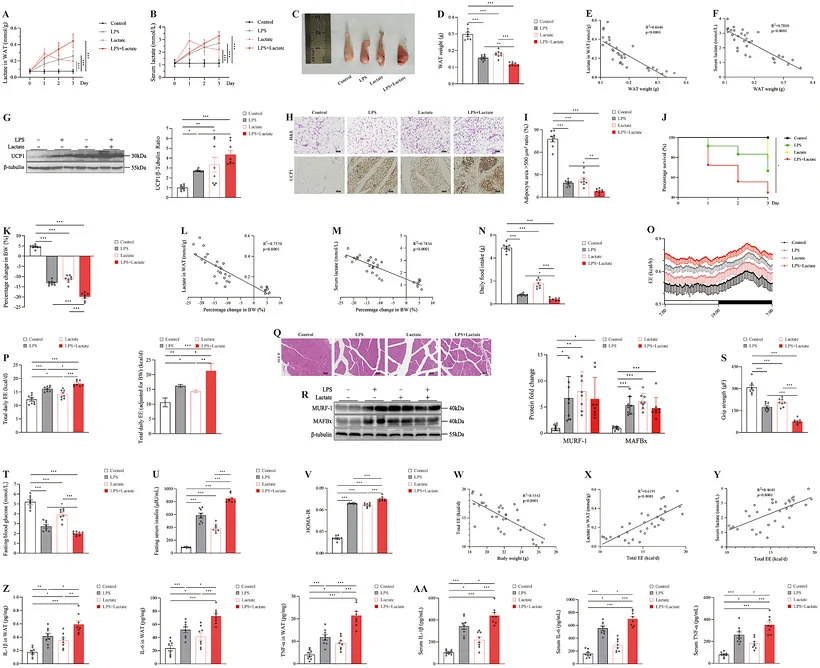
Exogenous lactate supplementation synergistically aggravated LPS‐induced WAT browning and sepsis‐associated metabolic disorders in mice. (A,B) Lactate concentrations in WAT and serum were measured via lactate assay kits at 24, 48, and 72 h after intraperitoneal injection of saline, lactate (0.5 g/kg), LPS (5 mg/kg), or LPS (5 mg/kg) plus lactate (0.5 g/kg) (*n* = 8 per group). (C) Representative images of WAT isolated from the inguinal region. (D) WAT weight. (E,F) Linear regression analyses evaluating associations between lactate concentrations in WAT and serum and WAT weight. (G) Western blot analysis of UCP1 in WAT. (H) Representative images of H&E staining and UCP1 immunohistochemistry in WAT sections. (I) Quantification of the proportion of adipocytes with a cross‐sectional area > 500 µm^2^. (J) Kaplan‐Meier survival curve depicting cumulative 72‐h overall survival. (K) Percentage change in BW. (L,M) Linear regression analyses evaluating associations between lactate concentrations in WAT and serum and percentage change in BW. (N) Daily food intake. (O,P) 24‐h EE curve and total daily EE (unadjusted data and data adjusted for body weight by ANCOVA) recorded by metabolic cages. (Q) Representative images of H&E staining in EDL sections. (R) Western blot analysis of MURF‐1 and MAFBx in EDL. (S) Forelimb grip strength measurement. (T–V) Fasting blood glucose, fasting serum insulin, and calculated HOMA‐IR for insulin resistance assessment. (W) Linear regression analyses evaluating associations between total EE and body weight. (X,Y) Linear regression analyses evaluating associations between lactate concentrations in WAT and serum and total EE. (Z‐AA) The levels of IL‐1β, IL‐6, and TNF‐α in WAT and serum were measured via ELISA. Data are expressed as mean ± SEM. ^*^ *p* < 0.05, ^**^ *p* < 0.01, ^***^ *p* < 0.001. WAT, white adipose tissue; LPS, lipopolysaccharide; UCP1, uncoupling protein 1; BW, body weight; EE, energy expenditure; H&E, Hematoxylin‐eosin; MURF‐1, muscle ring‐finger‐1; MAFBx, muscle atrophy F‐Box; EDL, extensor digitorum longus; HOMA‐IR, homeostatic model assessment of insulin resistance; IL‐1β, interleukin‐1 beta; IL‐6, interleukin‐6; TNF‐α, tumor necrosis factor‐alpha.

Prior reports have demonstrated that intraperitoneal (i.p.) injection of oxamate or 2‐DG could suppress lactate production in mice (Figure 4W) [19, 27]. We consistently observed that i.p. injection of oxamate or 2‐DG suppressed endogenous lactate production both in serum and WAT (Figure 4A,B). However, genetically deleting UCP1 did not rescue LPS‐induced lactate accumulation (Figure S2A,B). Histological and morphological analyses showed that pharmacological suppression of lactate production significantly attenuated LPS‐induced adipose atrophy and multilocular lipid droplet formation (Figure 4C–F). Western blot analysis and immunohistochemistry showed that pretreatment with either inhibitor attenuated LPS‐induced upregulation of UCP1 expression (Figure 4E,G). Suppressing lactate production attenuated LPS‐induced body weight loss, reduced food intake, and increased EE (Figure 4K–N). LPS‐induced skeletal muscle atrophy, muscle dysfunction, insulin resistance, and inflammatory response were similarly attenuated with the administration of oxamate or 2‐DG (Figure 4O–V).

**FIGURE 4:**
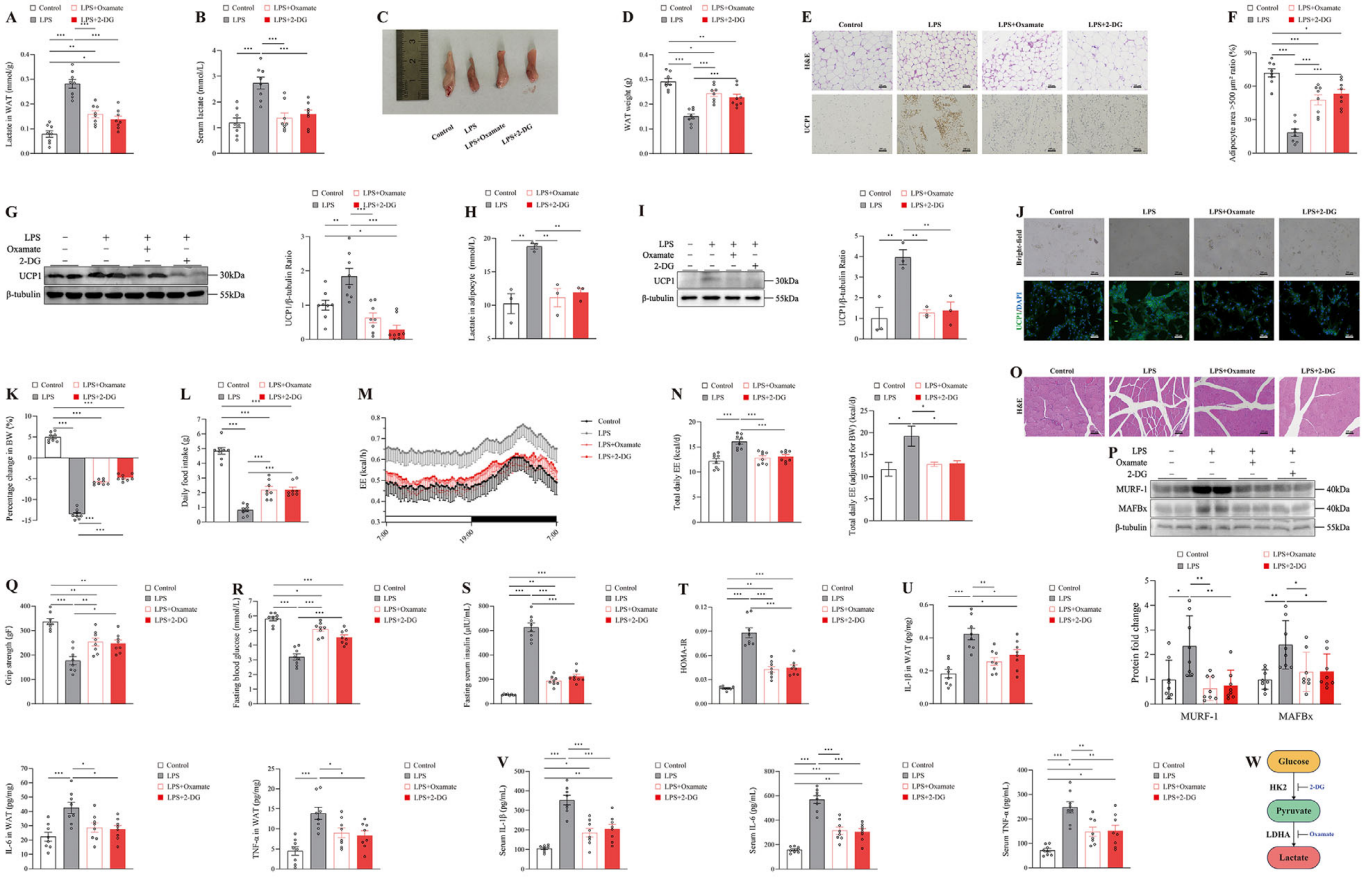
Pharmacological blockade of endogenous lactate production attenuated LPS‐induced WAT browning and systemic metabolic disorders in mice and SVF‐derived adipocytes. (A,B) Lactate concentrations in WAT and serum were measured via lactate assay kits at 72 h after intraperitoneal injection of saline, LPS (5 mg/kg), oxamate (0.75 g/kg/d, given 3 h prior to LPS), or 2‐DG (0.5 g/kg/d, given 3 h prior to LPS) (*n* = 8 per group). (C) Representative images of WAT isolated from the inguinal region. (D) WAT weight. (E) Representative images of H&E staining and UCP1 immunohistochemistry in WAT sections. (F) Quantification of the proportion of adipocytes with a cross‐sectional area > 500 µm^2^. (G) Western blot analysis of UCP1 in WAT. (H–J) SVF‐derived adipocytes were pre‐treated with oxamate (20 mM) or 2‐DG (10 mM) for 1 h prior to LPS (1 µg/mL) stimulation (*n* = 3 biologically independent samples in each group). Intracellular lactate concentrations were measured via lactate assay kits at 24 h after LPS stimulation. Western blot analysis of UCP1 in adipocytes. And representative images of bright‐field and UCP1 immunofluorescence in adipocytes. (K–N) Metabolic parameters continuously recorded by metabolic cages: percentage change in BW, daily food intake, 24‐h EE curve, and total daily EE (unadjusted data and data adjusted for body weight by ANCOVA). (O) Representative images of H&E staining in EDL sections. (P) Western blot analysis of MURF‐1 and MAFBx in EDL. (Q) Forelimb grip strength measurement. (R–T) Fasting blood glucose, fasting serum insulin, and calculated HOMA‐IR for insulin resistance assessment. (U‐V) The levels of IL‐1β, IL‐6, and TNF‐α in WAT and serum were measured via ELISA. (W) Schematic illustration of lactate production inhibition by oxamate and 2‐DG. Data are expressed as mean ± SEM. ^*^ *p* < 0.05, ^**^ *p* < 0.01, ^***^ *p* < 0.001. WAT, white adipose tissue; LPS, lipopolysaccharide; H&E, Hematoxylin‐eosin; UCP1, uncoupling protein 1; BW, body weight; EE, energy expenditure; SVF, stromal vascular fraction; MURF‐1, muscle ring‐finger‐1; MAFBx, muscle atrophy F‐Box; EDL, extensor digitorum longus; HOMA‐IR, homeostatic model assessment of insulin resistance; IL‐1β, interleukin‐1 beta; IL‐6, interleukin‐6; TNF‐α, tumor necrosis factor‐alpha.

In vitro experiments, we first verified whether lactate could induce SVF‐differentiated adipocytes browning. Western blot analysis and immunofluorescence showed significant UCP1 upregulation in adipocytes following lactate stimulation. Meanwhile, morphological imaging detected lactate‐induced adipocyte atrophy (Figure S3). Subsequently, SVF‐differentiated adipocytes were treated with serially diluted LPS to identify the optimal stimulatory concentration for subsequent cellular experiments. Western blot analysis and immunofluorescence showed robust UCP1 upregulation in adipocytes exposed to 1.0 µg/mL LPS. Morphological imaging also detected severe adipocyte atrophy at this dosage (Figure S4). Consistent with in vivo findings, oxamate or 2‐DG treatment suppressed intracellular lactate production, inhibited the expression of UCP1, and rescued LPS‐induced adipocyte atrophy (Figure 4H–J).

Collectively, these in vivo and in vitro data demonstrated that sustained lactate accumulation acted as a key upstream pathogenic driver for pathological WAT browning and subsequent systemic metabolic disorders during sepsis.

### 2.5 Lactate Drives WAT Browning to Aggravate Sepsis‐Associated Metabolic Disorders in a GPR81‐Dependent Manner

To identify the specific cell‐surface monocarboxylate transporters (MCTs) or lactate receptor expressed on adipocytes, SVF‐derived adipocytes from WT mice were individually transfected with siRNAs targeting MCT1, MCT4, GPR4, GPR81, and GPR132 before LPS stimulation. LPS stimulation increased lactate accumulation in adipocytes across all siRNA‐transfected adipocyte groups (Figure S5A). Western blot analysis and immunofluorescence showed that only GPR81 siRNA transfection significantly attenuated LPS‐induced upregulation of UCP1 expression, whereas MCT1, MCT4, GPR4, and GPR132 siRNAs transfection exerted no inhibitory effect on UCP1 expression (Figure S5B,C). Morphological imaging further confirmed GPR81 siRNA transfection attenuated LPS‐induced adipocyte atrophy (Figure S5C).

To further validate whether lactate exerted metabolic disorders in a GPR81‐dependent manner, we systematically compared WT septic mice and GPR81 knockout (GPR81^−/−^) septic mice. No significant differences in lactate accumulation in WAT and serum were detected between GPR81^−/−^ mice and WT mice after LPS injection (Figure 5A,B). Genetically deleting GPR81 attenuated LPS‐induced adipose atrophy and multilocular lipid droplet formation (Figure 5D–G). Western blot analysis and immunohistochemistry showed that genetically deleting GPR81 significantly attenuated LPS‐induced upregulation of UCP1 (Figure 5C,D). Genetically deleting GPR81 also rescued LPS‐induced negative energy balance (Figure 5K–N). Meanwhile, LPS‐induced skeletal muscle atrophy, muscle dysfunction, insulin resistance, and inflammatory response were attenuated in GPR81^−/−^ septic mice (Figure 5O–V). Although no significant difference in intracellular lactate accumulation was found between GPR81^−/−^ SVF‐derived adipocytes and WT SVF‐derived adipocytes, genetically reduced UCP1 expression rescued adipocyte atrophy (Figure 5H–J).

**FIGURE 5:**
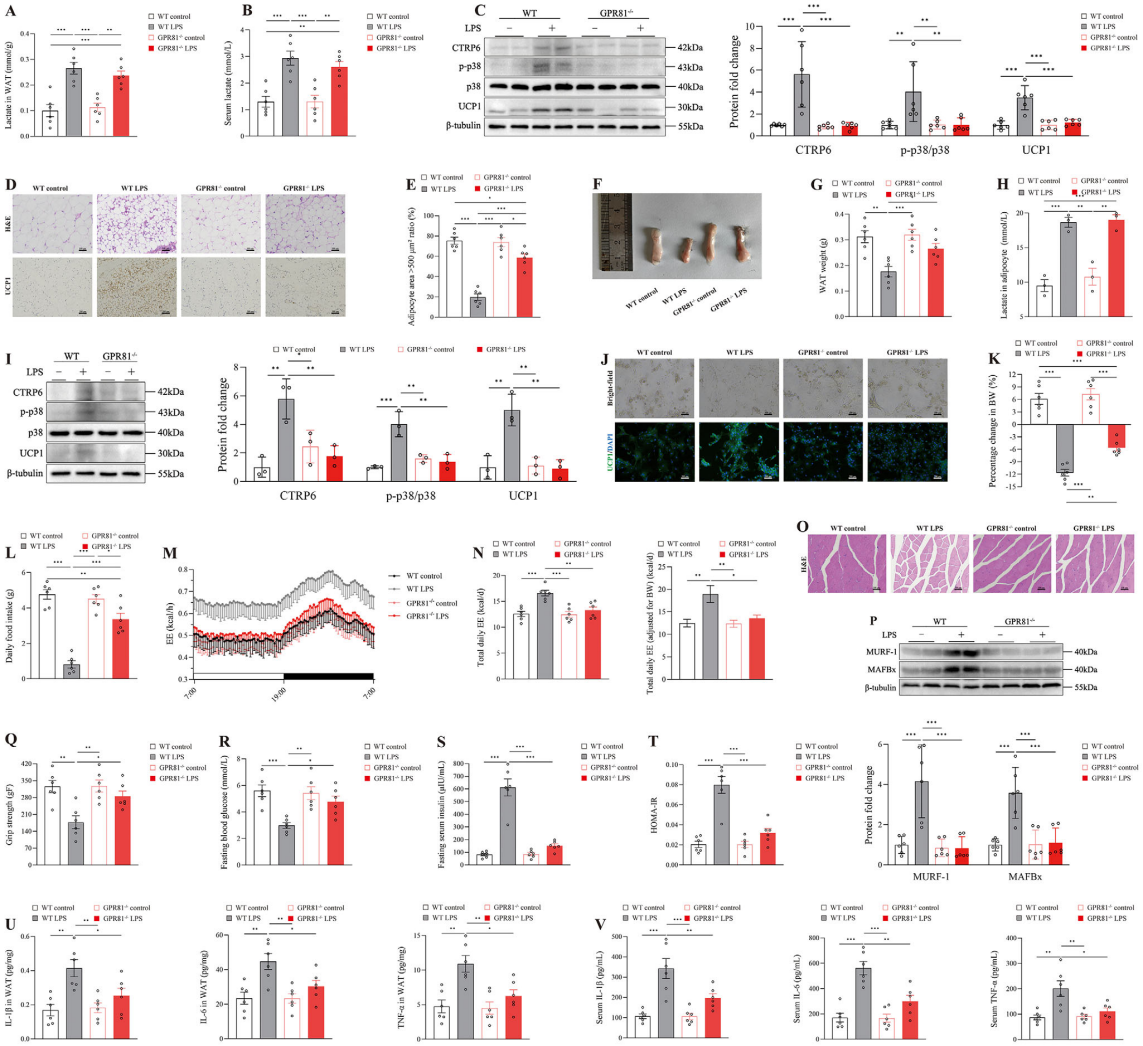
Genetically deleting GPR81 attenuated LPS‐induced pathological WAT browning and systemic metabolic disorders in mice and SVF‐derived adipocytes. (A,B) Lactate concentrations in WAT and serum were measured via lactate assay kits at 72 h after intraperitoneal injection of saline or LPS (5 mg/kg) in WT and GPR81^−/−^ mice (*n* = 6 per group). (C) Western blot analysis of CTRP6, p‐p38, and UCP1 in WAT. (D) Representative images of H&E staining and UCP1 immunohistochemistry in WAT sections. (E) Quantification of the proportion of adipocytes with a cross‐sectional area > 500 µm^2^. (F) Representative images of WAT isolated from the inguinal region. (G) WAT weight. (H–J) WT SVF‐derived adipocytes and GPR81^−/−^ SVF‐derived adipocytes were treated with LPS (1 µg/mL) (*n* = 3 biologically independent samples in each group). Intracellular lactate concentrations were measured via lactate assay kits at 24 h after LPS stimulation. Western blot analysis of CTRP6, p‐p38, and UCP1 in adipocytes. Representative images of bright‐field and UCP1 immunofluorescence in adipocytes. (K‐N) Metabolic parameters continuously recorded by metabolic cages: percentage change in BW, daily food intake, 24‐h EE curve, and total daily EE (unadjusted data and data adjusted for body weight by ANCOVA). (O) Representative images of H&E staining in EDL sections. (P) Western blot analysis of MURF‐1 and MAFBx in EDL. (Q) Forelimb grip strength measurement. (R–T) Fasting blood glucose, fasting serum insulin, and calculated HOMA‐IR for insulin resistance assessment. (U,V) The levels of IL‐1β, IL‐6, and TNF‐α in WAT and serum were measured via ELISA. Data are expressed as mean ± SEM. ^*^ *p* < 0.05, ^**^ *p* < 0.01, ^***^ *p* < 0.001. WAT, white adipose tissue; LPS, lipopolysaccharide; WT, wild‐type; SVF, stromal vascular fraction; GPR81, G‐protein‐coupled receptor 81; CTRP6, C1q/tumor necrosis factor‐related protein 6; UCP1, uncoupling protein 1; H&E, Hematoxylin‐eosin; BW, body weight; EE, energy expenditure; EDL, extensor digitorum longus; MURF‐1, muscle ring‐finger‐1; MAFBx, muscle atrophy F‐Box; HOMA‐IR, homeostatic model assessment of insulin resistance; IL‐1β, interleukin‐1 beta; IL‐6, interleukin‐6; TNF‐α, tumor necrosis factor‐alpha.

Given that global GPR81 knockout cannot fully clarify whether the protective phenotype originates specifically from WAT, we established an adeno‐associated virus serotype 9 (AAV9)‐mediated adipose‐specific GPR81 knockdown mouse model, followed by LPS administration. No significant differences in lactate accumulation in WAT and serum were detected between LPS and AAV9‐miR‐GPR81 + LPS groups (Figure 6A,B). Adipose‐specific GPR81 knockdown attenuated LPS‐induced upregulation of UCP1, adipocyte atrophy, and multilocular lipid droplet formation (Figure 6C–G). Adipose‐specific GPR81 knockdown also rescued LPS‐induced negative energy balance (Figure 6H–K). Meanwhile, LPS‐induced skeletal muscle atrophy, muscle dysfunction, insulin resistance, and inflammatory response were attenuated in the AAV9‐miR‐GPR81 + LPS group (Figure 6L–S).

**FIGURE 6:**
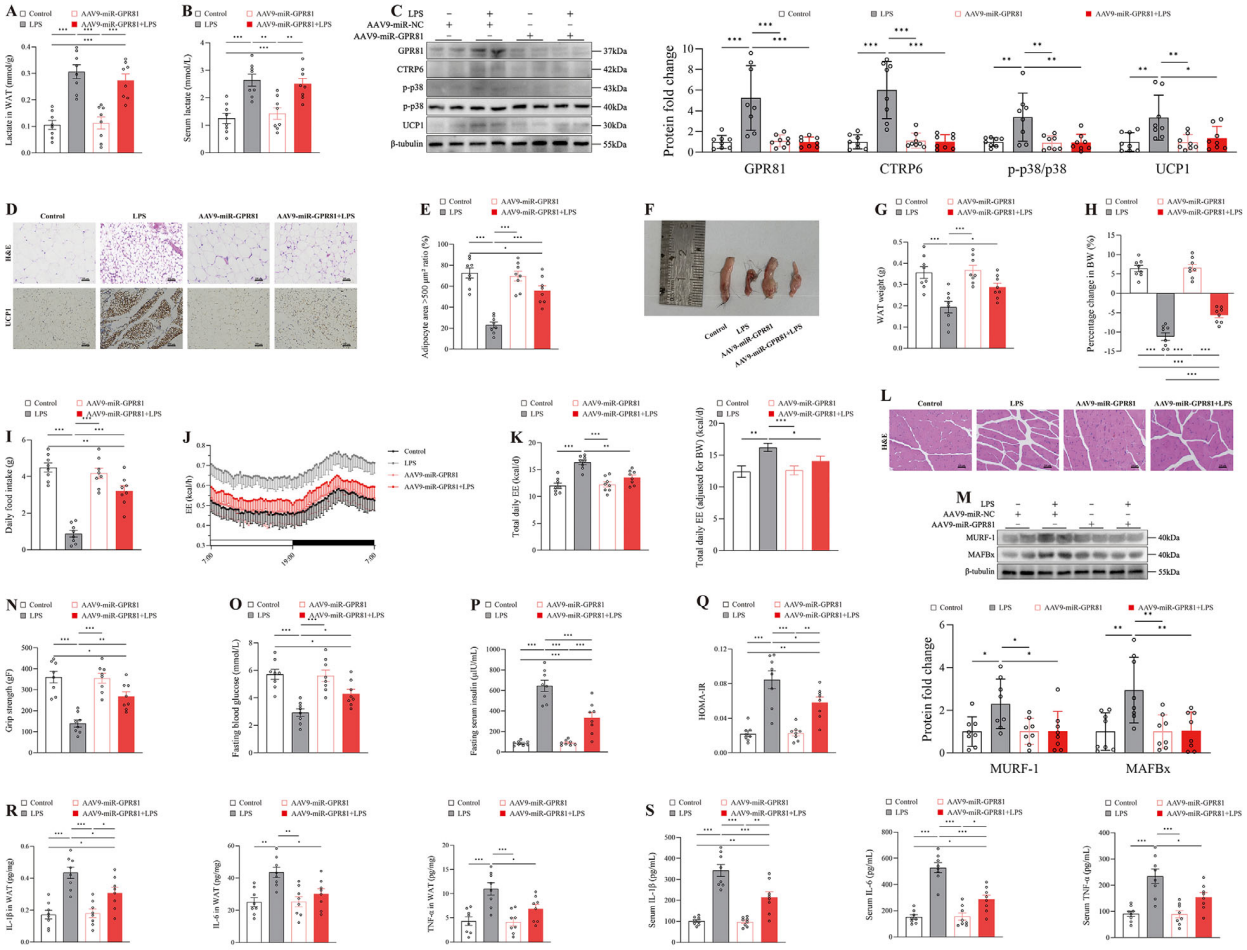
Adipose‐specific GPR81 knockdown attenuated LPS‐induced pathological WAT browning and systemic metabolic disorders in mice. (A,B) Local AAV9‐miR‐GPR81 and AAV9‐miR‐NC were delivered into bilateral inguinal WAT (50 µL per side, 100 µL per mouse); 3 weeks after AAV9 injection, mice were intraperitoneally injected with saline or LPS (5 mg/kg), and lactate concentrations in WAT and serum were measured via lactate assay kits at 72 h after intraperitoneal injection of saline or LPS (*n* = 8 per group). (C) Western blot analysis of GPR81, CTRP6, p‐p38, and UCP1 in WAT. (D) Representative images of H&E staining and UCP1 immunohistochemistry in WAT sections. (E) Quantification of the proportion of adipocytes with a cross‐sectional area > 500 µm^2^. (F) Representative images of WAT isolated from the inguinal region. (G) WAT weight. (H–K) Metabolic parameters continuously recorded by metabolic cages: percentage change in BW, daily food intake, 24‐h EE curve, and total daily EE (unadjusted data and data adjusted for body weight by ANCOVA). (L) Representative images of H&E staining in EDL sections. (M) Western blot analysis of MURF‐1 and MAFBx in EDL. (N) Forelimb grip strength measurement. (O–Q) Fasting blood glucose, fasting serum insulin, and calculated HOMA‐IR for insulin resistance assessment. (R,S) The levels of IL‐1β, IL‐6, and TNF‐α in WAT and serum were measured via ELISA. Data are expressed as mean ± SEM. ^*^ *p* < 0.05, ^**^ *p* < 0.01, ^***^ *p* < 0.001. AAV9, adeno‐associated virus serotype 9; WAT, white adipose tissue; LPS, lipopolysaccharide; GPR81, G‐protein‐coupled receptor 81; CTRP6, C1q/tumor necrosis factor‐related protein 6; UCP1, uncoupling protein 1; H&E, Hematoxylin‐eosin; BW, body weight; EE, energy expenditure; EDL, extensor digitorum longus; MURF‐1, muscle ring‐finger‐1; MAFBx, muscle atrophy F‐Box; HOMA‐IR, homeostatic model assessment of insulin resistance; IL‐1β, interleukin‐1 beta; IL‐6, interleukin‐6; TNF‐α, tumor necrosis factor‐alpha.

Collectively, these in vitro and in vivo knockout data demonstrated that lactate drove pathological WAT browning in a GPR81‐dependent manner, thereby resulting in a series of sepsis‐associated metabolic disorders.

### 2.6 CTRP6 and p38 Function as Downstream Mediators of GPR81 to Mediate LPS‐Induced WAT Browning and Metabolic Disorders

To systematically validate whether CTRP6 and p38 served as key downstream mediators through which GPR81 signaling drove WAT browning and metabolic disorders, we conducted AAV9‐mediated overexpression of CTRP6 or MKK6 in GPR81^−/−^ mice and pharmacological modulation of CTRP6 expression (rosiglitazone, a PPARγ agonist reported to downregulate CTRP6 expression [28]) or p‐p38 activity (SB203580) in WT septic mice.

No significant differences in lactate accumulation in serum and WAT were detected after AAV9‐mediated overexpression of CTRP6 or MKK6 in GPR81^−/−^ mice (Figure 7A,B). AAV9‐CTRP6 significantly upregulated the protein expression of CTRP6, and AAV9‐MKK6 increased p‐p38 in WAT from GPR81^−/−^ mice. In the absence of LPS challenge, both interventions were sufficient to induce UCP1 upregulation in the WAT of GPR81^−/−^ mice (Figure 7C,D). AAV9‐mediated overexpression of CTRP6 or MKK6 led to adipose atrophy and multilocular lipid droplet formation in GPR81^−/−^ mice (Figure 7D–G). Overexpression of CTRP6 or MKK6 suppressed body weight gain and markedly increased EE in GPR81^−/−^ mice (Figure 7H–J). For skeletal muscle phenotypes, overexpression of CTRP6 or MKK6 aggravated myofiber atrophy, upregulated MURF‐1 and MAFBx protein expression, and impaired forelimb grip strength in GPR81^−/−^ mice (Figure 7K–M). Glucose homeostasis was disrupted, and the HOMA‐IR was increased after CTRP6 or MKK6 overexpression (Figure 7N–P). Overexpression of CTRP6 or MKK6 also increased the levels of inflammatory cytokines in WAT and serum (Figure 7Q,R).

**FIGURE 7:**
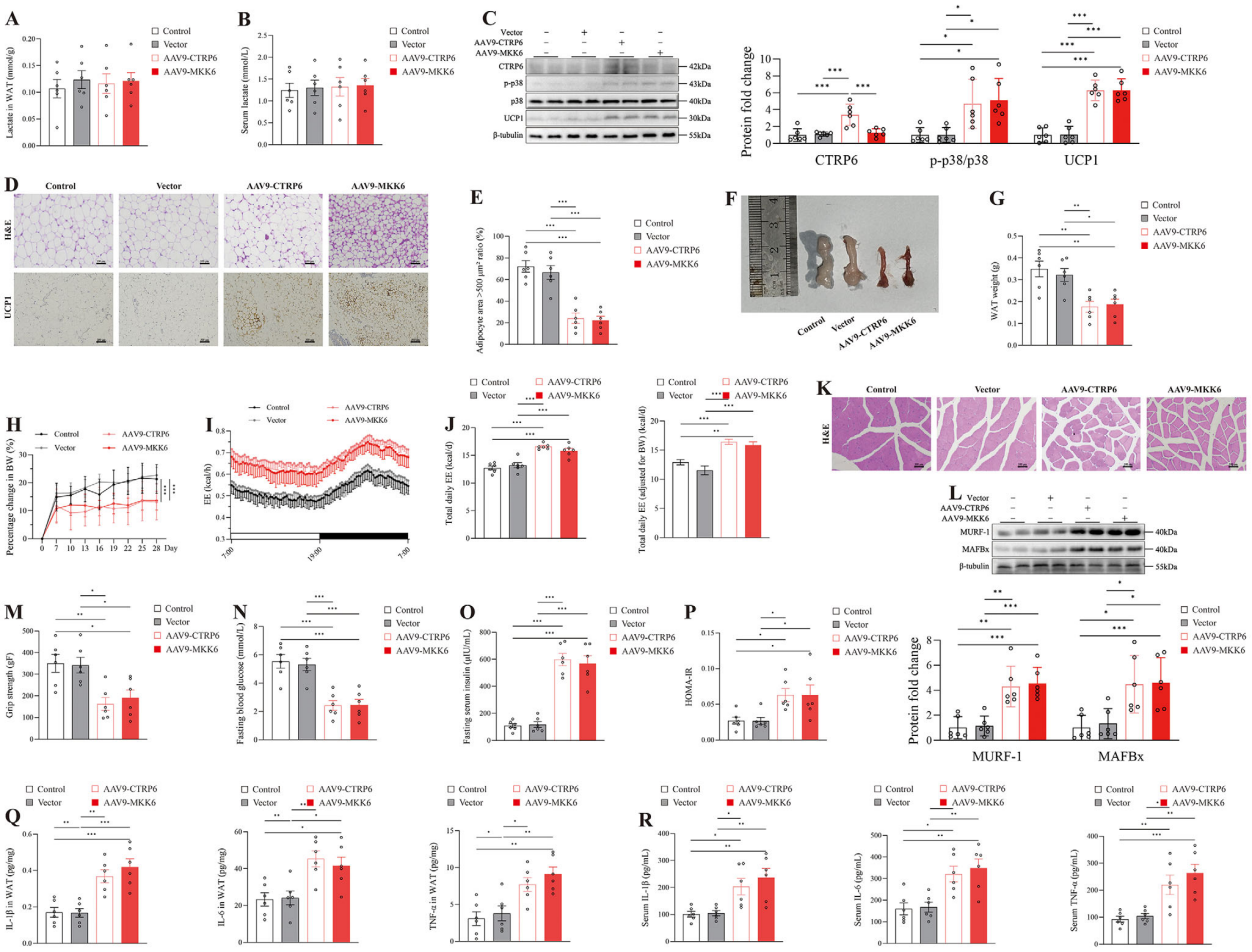
AAV9‐mediated CTRP6 overexpression or MKK6‐mediated p38 activation aggravated WAT browning and systemic metabolic disorders in GPR81^−/−^ mice. (A,B) Local AAV9 delivery into bilateral inguinal WAT (50 µL per side, 100 µL per mouse); lactate concentrations in WAT and serum were measured via lactate assay kits 28 days post‐injection (*n* = 6 per group). (C) Western blot analysis of CTRP6, p‐p38, and UCP1 in WAT. (D) Representative images of H&E staining and UCP1 immunohistochemistry in WAT sections. (E) Quantification of the proportion of adipocytes with a cross‐sectional area > 500 µm^2^. (F) Representative images of WAT isolated from the inguinal region. (G) WAT weight. (H) Relative body weight fluctuations monitored over the 4 weeks post‐viral injection. (I,J) Metabolic parameters continuously recorded by metabolic cages: 24‐h EE curve and total daily EE (unadjusted data and data adjusted for body weight by ANCOVA) on day 28 after injection. (K) Representative images of H&E staining in EDL sections. (L) Western blot analysis of MURF‐1 and MAFBx in EDL. (M) Forelimb grip strength measurement. (N–P) Fasting blood glucose, fasting serum insulin, and calculated HOMA‐IR for insulin resistance assessment. (Q,R) The levels of IL‐1β, IL‐6, and TNF‐α in WAT and serum were measured via ELISA. Data are expressed as mean ± SEM. ^*^ *p* < 0.05, ^**^ *p* < 0.01, ^***^ *p* < 0.001. AAV9, adeno‐associated virus serotype 9; WAT, white adipose tissue; LPS, lipopolysaccharide; WT, wild‐type; GPR81, G‐protein‐coupled receptor 81; CTRP6, C1q/tumor necrosis factor‐ related protein 6; UCP1, uncoupling protein 1; H&E, Hematoxylin‐eosin; BW, body weight; EE, energy expenditure; EDL, extensor digitorum longus; MURF‐1, muscle ring‐finger‐1; MAFBx, muscle atrophy F‐Box; HOMA‐IR, homeostatic model assessment of insulin resistance; IL‐1β, interleukin‐1 beta; IL‐6, interleukin‐6; TNF‐α, tumor necrosis factor‐alpha.

We next used pharmacological agents to reduce CTRP6 expression or to inhibit p‐p38 activity in WT septic mice. Pretreatment with rosiglitazone, which has been reported to downregulate CTRP6 expression [28], or SB203580 (p‐p38 inhibitor) exerted no obvious effect on lactate accumulation in WAT and serum (Figure 8A,B). Rosiglitazone reduced CTRP6 expression, and SB203580 inhibit p‐p38 activity in WAT, and both treatments attenuated LPS‐induced upregulation of corresponding proteins and UCP1 in WAT (Figure 8C,D). Histological and morphological detection of WAT showed that rosiglitazone or SB203580 treatment significantly attenuated LPS‐induced adipose atrophy and multilocular lipid droplet formation (Figure 8D–G). Rosiglitazone or SB203580 treatment effectively attenuated LPS‐induced weight loss, anorexia, and increased EE (Figure 8K–N). Furthermore, Rosiglitazone or SB203580 treatment attenuated skeletal muscle atrophy, inhibited the expression of muscle atrophy markers, and preserved forelimb grip strength (Figure 8O–Q). These inhibitory interventions also restored glucose metabolism and attenuated insulin resistance in septic mice (Figure 8R–T). Rosiglitazone or SB203580 treatment attenuated the upregulation of inflammatory cytokines in WAT and serum (Figure 8U,V).

**FIGURE 8:**
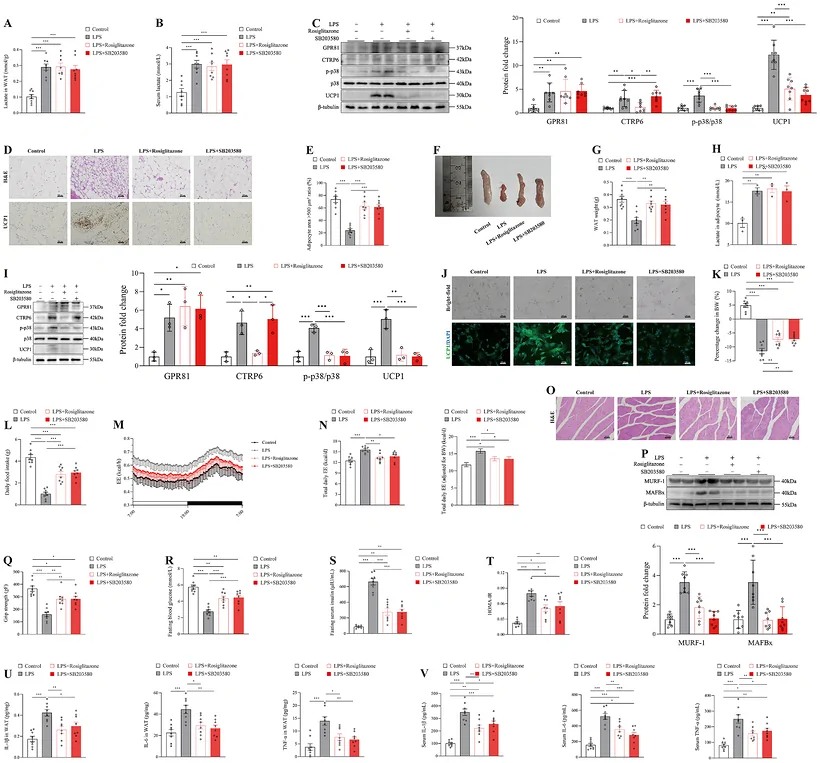
Pharmacological downregulation of CTRP6 or inhibition of p‐p38 attenuated LPS‐induced WAT browning and systemic metabolic disorders in mice and SVF‐derived adipocytes. (A,B) Lactate concentrations in WAT and serum were measured via lactate assay kits at 72 h after intraperitoneal injection of saline, LPS (5 mg/kg), rosiglitazone (15 mg/kg/d, given 3 h prior to LPS), or SB203580 (20 mg/kg/d, given 3 h prior to LPS) (*n* = 8 per group). (C) Western blot analysis of GPR81, CTRP6, p‐p38, and UCP1 in WAT. (D) Representative images of H&E staining and UCP1 immunohistochemistry in WAT sections. (E) Quantification of the proportion of adipocytes with a cross‐sectional area > 500 µm^2^. (F) Representative images of WAT isolated from the inguinal region. (G) WAT weight. (H‐J) SVF‐derived adipocytes were pre‐treated with rosiglitazone (10 µm) or SB203580 (10 µm) for 1 h prior to LPS (1 µg/mL) stimulation (*n* = 3 biologically independent samples in each group). Intracellular lactate concentrations were measured via lactate assay kits at 24 h after LPS stimulation. Western blot analysis of GPR81, CTRP6, p‐p38, and UCP1 in adipocytes. Representative images of bright‐field and UCP1 immunofluorescence in adipocytes. (K–N) Metabolic parameters continuously recorded by metabolic cages: percentage change in BW, daily food intake, 24‐h EE curve, and total daily EE (unadjusted data and data adjusted for body weight by ANCOVA). (O) Representative images of H&E staining in EDL sections. (P) Western blot analysis of MURF‐1 and MAFBx in EDL. (Q) Forelimb grip strength measurement. (R–T) Fasting blood glucose, fasting serum insulin, and calculated HOMA‐IR for insulin resistance assessment. (U,V) The levels of IL‐1β, IL‐6, and TNF‐α in WAT and serum were measured via ELISA. Data are expressed as mean ± SEM. ^*^ *p* < 0.05, ^**^ *p* < 0.01, ^***^ *p* < 0.001. WAT, white adipose tissue; LPS, lipopolysaccharide; SVF, stromal vascular fraction; GPR81, G‐protein‐coupled receptor 81; CTRP6, C1q/tumor necrosis factor‐ related protein 6; UCP1, uncoupling protein 1; H&E, Hematoxylin‐eosin; BW, body weight; EE, energy expenditure; EDL, extensor digitorum longus; MURF‐1, muscle ring‐finger‐1; MAFBx, muscle atrophy F‐Box; HOMA‐IR, homeostatic model assessment of insulin resistance; IL‐1β, interleukin‐1 beta; IL‐6, interleukin‐6; TNF‐α, tumor necrosis factor‐alpha.

In cellular experiments, rosiglitazone or SB203580 did not lower LPS‐induced intracellular lactate concentration in adipocytes (Figure 8H). In contrast, rosiglitazone or SB203580 treatment significantly attenuated the LPS‐induced upregulation of corresponding proteins and UCP1 protein expression, and rescued adipocyte atrophy (Figure 8I–J).

To further validate CTRP6 and p‐p38 as important downstream mediators connecting GPR81 activation to LPS‐induced pathological WAT browning, we integrated all preceding in vivo and in vitro data. Western blot analysis showed concurrent upregulation of GPR81, CTRP6, p‐p38, and UCP1 in WAT isolated from septic patients (Figure 1Q). LPS injection significantly upregulated the expression of GPR81, CTRP6, and p‐p38 in WAT from both WT and UCP1^−/−^ mice (Figure S2C). Co‐administration of LPS and lactate exerted synergistic inductive effects on GPR81, CTRP6, and p‐p38 (Figure S6). Consistent in vivo and in vitro data demonstrated that genetically deleting global GPR81 and adipose‐specific GPR81 knockdown significantly attenuated LPS‐induced upregulation of CTRP6 and p‐p38 (Figures 5C,I and 6C). Pharmacological suppression of lactate production via oxamate or 2‐DG pretreatment markedly reduced LPS‐induced upregulation of GPR81, CTRP6, and p‐p38 (Figure S7).

Collectively, integrated clinical, animal and cellular data comprehensively demonstrated that lactate drove pathological WAT browning and subsequent sepsis‐associated metabolic disorders through a GPR81‐dependent signaling pathway that involved CTRP6 and p‐p38.

## 3 Discussion

Sepsis‐associated metabolic disorders represent a major unmet clinical challenge in critical care [29]. Current standard treatments that center on anti‐infection and organ support effectively suppress inflammatory cascades, but fail to reverse persistent metabolic disorders, including proteolysis, sustained anorexia, and insulin resistance [30]. These metabolic complications directly contribute to long‐term physical impairment and elevated post‐discharge mortality among sepsis survivors [31]. Although lactate is a well‐established prognostic biomarker for sepsis, the full signaling pathway through which lactate mediates sepsis‐induced metabolic dysfunction remains incompletely characterized [32]. Lactate‐induced WAT browning was first described in burn injury and cancer cachexia [13, 21]. Our prior work first linked this pathway to sepsis‐associated metabolic disorders, but did so through a nutritional intervention that reduced lactate, GPR81, CTRP6, and p‐p38 in parallel, and therefore could not establish causality, the adipocyte as the causal cell type, or human relevance [24]. In the present study, we adopted a multi‐dimensional research strategy integrating clinical data, in vivo animal functional interventions, and in vitro primary adipocyte experiments to delineate this full pathogenic signaling pathway. Mechanistically, sepsis‐induced lactate accumulation facilitates lactate binding to GPR81, which sequentially upregulates CTRP6 and p‐p38, ultimately increasing UCP1 expression to drive pathological WAT browning and consequently aggravate sepsis‐associated metabolic disorders. Our findings uncover the molecular link between hyperlactatemia and metabolic disorders, and identify potential targets that warrant further investigation for sepsis‐associated metabolic disorders refractory to conventional anti‐infective and nutritional support treatments.

Clinically, lactate has long been recognized as a prognostic biomarker of tissue hypoperfusion and mortality risk in septic patients, yet its metabolic regulatory functions in adipose tissue remain understudied [33, 34]. Our clinical data demonstrated that lactate accumulation and pathological WAT browning were closely correlated with metabolic disorders in septic patients. Quantitative CT analysis at the L3 vertebral level detected substantially greater depletion of TAMT, SAT, and VAT in septic patients. Linear regression correlation analysis further confirmed that higher lactate concentrations in serum and WAT correlated with severe muscle and adipose tissue wasting. Targeted UHPLC‐MRM‐MS metabolomics profiling of omental WAT revealed aberrantly upregulated glycolysis and lactate metabolic pathways in septic patients. Notably, septic patients exhibited distinct reddish‐brown morphological changes in omental WAT, accompanied by abundant multilocular lipid droplets and elevated UCP1 expression, all of which revealed significant WAT browning during the stress period. The close correlation between lactate concentration and the severity of WAT browning further supports that lactate acts as a bioactive metabolic mediator that drives WAT browning, rather than a passive glycolytic end‐product. Earlier preclinical studies using burn injury and cancer cachexia models only demonstrated that lactate could trigger adipocyte thermogenic programs; our clinical data confirms concurrent lactate overload and pathological WAT browning in septic patients, establishing a robust clinical basis for delineating the downstream signaling pathway initiated by lactate.

Prior to mechanistic investigation, we systematically evaluated four widely used sepsis models in mice, including intraperitoneal injection of LPS, CLP, intraperitoneal injection of *S. aureus*, and intranasal instillation of *S. aureus*. Although CLP and *S. aureus*‐infection models better mimicked clinical polymicrobial sepsis, these models displayed marked inter‐individual heterogeneity reflected by high CV for core metabolic indicators, which would confound the interpretation of adipose‐centric phenotypes. Therefore, given its superior reproducibility of metabolic indicators, the LPS‐induced sepsis model was selected for all subsequent mechanistic investigations. UCP1 serves as the core functional marker that defines pathological WAT browning [35]. In burn injury, cancer cachexia, and other chronic inflammatory diseases, persistent inflammatory stimulation drives sustained UCP1 upregulation, which in turn aggravates excessive EE [36]. Previous sepsis‐related studies also reported increased UCP1 expression in WAT isolated from septic mice and critically ill patients [12, 36]. However, none of these prior studies definitively established whether UCP1 was required to activate systemic metabolic disorders. In the present study, genetically deleting UCP1 significantly attenuated LPS‐induced metabolic disorders including negative energy balance, muscle atrophy, and insulin resistance in septic mice. Under acute septic stress, widespread reprogramming of systemic glucose metabolism drives robust lactate overproduction independent of tissue hypoxia in septic patients [34]. Robust inflammatory activation drives systemic metabolic reprogramming toward aerobic glycolysis. Meanwhile, sepsis‐induced glucocorticoid resistance impairs hepatic gluconeogenesis and reduces lactate clearance, ultimately causing sustained lactate accumulation throughout the body [18]. Importantly, lactate accumulating within WAT acts as a persistent local stimulus that continuously initiates adipocyte thermogenic reprogramming [37]. Notably, genetically deleting UCP1 failed to alter LPS‐induced lactate accumulation in both WAT and serum. Exogenous lactate supplementation aggravated LPS‐induced WAT browning and systemic metabolic disorders, whereas pharmacological suppression of endogenous lactate production with oxamate and 2‐DG attenuated both pathological phenotypes. Collectively, these data confirm that lactate accumulation precedes and drives UCP1‐mediated WAT browning, rather than hypermetabolism acting as a secondary driver of lactate overproduction.

Multiple MCTs and GPRs mediate lactate sensing and transmembrane transport in adipocytes; among them, MCT1, MCT4, GPR4, GPR81, and GPR132 exert distinct biological functions in response to lactate accumulation [38, 39, 40, 41]. In the present study, we identified GPR81 as the primary receptor responsible for pathological WAT browning. Our siRNA screening against a panel of candidate transporters and receptors demonstrated that only GPR81 silencing attenuated LPS‐induced UCP1 upregulation in SVF‐derived adipocytes, whereas knockdown of MCT1, MCT4, GPR4, and GPR132 produced no such inhibitory effect. GPR81^−/−^ mice exhibited prominent resistance to LPS‐induced adipose atrophy, excessive EE, and insulin resistance, further confirming that GPR81 plays a critical role in lactate's pathogenic actions. Considering that global GPR81 knockout abrogated GPR81 expression across all tissues and could not distinguish adipose‐specific effects from contributions of other organs, we further constructed an AAV9‐mediated adipose‐specific GPR81 knockdown mouse model. Adipose‐specific GPR81 knockdown also did not affect lactate concentrations in serum and WAT, yet it significantly attenuated LPS‐induced WAT browning and metabolic disorders. These findings showed that adipocyte‐expressed GPR81 mediated lactate‐induced pathological WAT browning, which drove downstream sepsis‐associated metabolic disorders. Prior obesity studies reported that GPR81‐mediated WAT browning relied on downstream p‐p38 activation, and pharmacological inhibition of p‐p38 markedly reduced lactate‐triggered UCP1 overexpression in adipocytes [42]. Consistent with these findings, a recent cachexia study also implicated a key GPR81‐p38 pathway in lactate‐mediated WAT browning [21]. Meanwhile, as a secreted C1q‐family adipokine, CTRP6 has been reported to upregulate p‐p38 expression and downstream pro‐inflammatory signaling in macrophages [43]. Existing studies have separately demonstrated the regulatory effects of GPR81 on adipocyte thermogenesis, p‐p38 on UCP1 transcription, and CTRP6 on inflammatory signaling, yet these molecules were investigated in isolated disease models without establishing a unified cascade during sepsis. Our multi‐level functional experiments further validated CTRP6 and p‐p38 as important downstream mediators that sequentially relay GPR81 signaling to drive UCP1‐dependent WAT browning. AAV9‐mediated overexpression and pharmacological intervention experiments validated the signaling pathway: overexpression of CTRP6 or MKK6 (which increased p‐p38) in GPR81^−/−^ mice is sufficient to drive UCP1 upregulation, WAT browning, and metabolic disorder even in the absence of septic insult; in contrast, rosiglitazone or SB203580 attenuated WAT browning and systemic metabolic disorders without changing lactate concentrations in WAT and serum. Importantly, because rosiglitazone is a PPARγ agonist with broad metabolic actions, its effects cannot be attributed solely to CTRP6; the target‐specific evidence for CTRP6 rests primarily on AAV9‐CTRP6 overexpression, whereas genetic loss‐of‐function evidence for CTRP6 is still lacking. Collectively, these data confirm that the lactate‐GPR81 pathway drives WAT browning, and functional evidence supports CTRP6‐p38 as an important downstream component of GPR81 signaling.

The lactate‐GPR81‐CTRP6‐p38 pathway uncovered in this present study resolves a prominent clinical puzzle regarding persistent metabolic disorders in septic patients. Sepsis‐induced lactate accumulation acts as a bioactive signaling molecule, not merely an inert glycolytic byproduct, and selectively binds GPR81 to initiate a downstream sequential signaling pathway centered on CTRP6 and p‐p38. This downstream effector module upregulates UCP1 expression, thereby driving pathological WAT browning to aggravate sepsis‐associated metabolic disorders. First‐line sepsis management consists of fluid resuscitation, antimicrobial agents, and organ function support [44]. Effective lactate clearance during sepsis therapy largely relies on preserved hepatic function [45]. From a physiological perspective, roughly 70% of circulating lactate is cleared by the liver, and an additional 30% by the kidneys [46]. However, sepsis induces excessive glycolysis and hepatic glucocorticoid resistance, which impairs hepatic gluconeogenesis and inhibits lactate clearance, ultimately leading to persistent systemic lactate accumulation [47]. Notably, even when standard interventions normalize circulating lactate, adipose tissue retains abnormally high lactate concentrations [48]. Collectively, these clinical and physiological findings explain why conventional clinical interventions fail to resolve metabolic disorder during the acute phase of sepsis. Integrating results from AAV9‐mediated overexpression and pharmacological intervention experiments, we demonstrated that the GPR81‐CTRP6‐p38 pathway acts as an important mediator linking hyperlactatemia to pathological WAT browning and sepsis‐associated metabolic disorders. Pharmacological intervention at the CTRP6 or p‐p38 node attenuated WAT browning and sepsis‐associated metabolic disorders, whereas forced expression of downstream signaling effectors was sufficient to induce these pathological phenotypes in GPR81^−/−^ mice even in the absence of septic insult. Taken together, these findings confirm that the GPR81‐CTRP6‐p38 pathway is an important determinant linking hyperlactatemia to sustained sepsis‐associated metabolic disorders, and identifies a potential target for attenuating metabolic disorders, pending validation of therapeutic efficacy in established sepsis.

This work has notable limitations that warrant consideration and point toward future research directions. First, our clinical cohort is limited by a relatively small sample size, which was driven by practical feasibility rather than a formal power calculation. Owing to this small sample size, univariate and multivariate regression analyses for confounder adjustment could not be performed, and statistical analyses were limited to univariate tests and simple linear regression; consequently, confounding by sepsis severity or organ dysfunction cannot be excluded. This small sample may reduce statistical power and generalizability, and the correlational findings should be interpreted with caution. All septic patients enrolled in our study developed sepsis secondary to cholelithiasis, as omental WAT could be readily harvested intraoperatively. By contrast, patients with sepsis originating from other primary infectious sources, including pneumonia, urinary tract infection, and soft tissue infection, were excluded from our study owing to the inability to obtain omental WAT. Since the intensity of inflammatory responses, immune function, and metabolic derangements differ markedly across infection types, follow‐up cohorts enrolling patients with diverse sepsis etiologies are needed to validate whether the lactate‐GPR81‐CTRP6‐p38 pathway exerts consistent regulatory effects on sepsis‐associated metabolic disorders across distinct infection backgrounds. Second, all animal experiments were performed exclusively in male mice. Given that sex hormones, particularly estrogen, are known to modulate adipose browning, UCP1 expression, and the inflammatory response to LPS, and that female mice generally exhibit a more resilient metabolic and inflammatory phenotype during sepsis, our findings may not be fully generalizable to female subjects. Future studies should validate the lactate‐GPR81‐CTRP6‐p38 pathway in female mice. Third, our animals were fed ad libitum and pair‐feeding experiments were not performed; consequently, the present data cannot quantitatively distinguish the independent contribution of sepsis‐induced anorexia from that of UCP1‐dependent thermogenesis to body‐weight loss and the catabolic phenotype. Although UCP1 deletion improved energy expenditure, insulin resistance, muscle atrophy, and inflammation, future pair‐fed studies will be required to define the exact contribution of reduced food intake. Body composition was not directly measured in the animal experiments because body weight and composition are themselves affected by the interventions. Fourth, all pharmacological and AAV9‐mediated interventions were administered before the septic insult. Our in vivo findings therefore demonstrate preventive effects rather than therapeutic efficacy against established sepsis, and whether targeting these nodes after sepsis onset attenuates established metabolic disorders remains untested. Future studies employing post‐sepsis treatment designs are needed to assess therapeutic potential. All mechanistic animal experiments used the LPS‐induced sepsis model. This model was chosen for its low variability and reproducible metabolic endpoints. However, this low variability partly reflects the artificial nature of a single bolus endotoxin stimulus, which does not fully recapitulate the polymicrobial and evolving pathophysiology of clinical sepsis. Our findings should therefore be interpreted with caution regarding clinical translatability, and confirmation in CLP or other polymicrobial models is warranted.

In summary, the previous study established this pathway in the context of a nutritional intervention [24]; the present study directly establishes the pathogenic causality of lactate‐driven adipose browning through human evidence, UCP1 loss‐of‐function, direct lactate perturbation, global GPR81 deletion, and adipose‐restricted GPR81 knockdown. This full pathogenic signaling pathway fills the gap between lactate accumulation and metabolic disorders during the stress period and identifies a potential therapeutic target that warrants evaluation in post‐sepsis treatment settings.

## 4 Materials and Methods

### 4.1 Human Experiment

All patients enrolled in this study were recruited between January 2025 and December 2025 at the Second Affiliated Hospital of Soochow University, China. Patient inclusion criteria were as follows: (1) cholelithiasis complicated with biliary tract infection requiring surgical intervention; (2) aged ≥ 18 years old; (3) provided written informed consent. Exclusion criteria included: (1) concomitant malignant tumors; (2) pre‐existing primary neuromuscular disorders; (3) long‐term bedridden status caused by cerebral infarction or other conditions; (4) pregnancy or lactation; (5) prior enrollment in other clinical trials. Patients were stratified into a sepsis group and a non‐sepsis group (*n* = 10 per group) according to the Sepsis‐3.0 diagnostic criteria [4]. Intraoperative serum and omental WAT were collected during surgical procedures. All patients underwent abdominal CT scans preoperatively and on postoperative day 3 to quantify changes in skeletal muscle, subcutaneous adipose, and visceral adipose tissue at the L3 vertebral level. This clinical study was approved by the Ethics Committee of Second Affiliated Hospital of Soochow University (JD‐LK2025192‐I01).

### 4.2 CT Image Acquisition and Processing

Cross‐sectional CT images were collected at the L3 vertebral level. Image analysis was performed using SliceOmatic Software 5.0 (TomoVision, USA). According to the standard Hounsfield unit (HU) range, tissue cross‐sectional areas of SAT (‐190 to −30 HU), VAT (−150 to −50 HU), and TAMT (−29 to 150 HU) were quantified [49].

### 4.3 Animals

WT C57BL/6 mice, GPR81^−/−^ mice (Strain S‐KO‐07425), and UCP1^−/−^ mice (Strain S‐KO‐05629) were purchased from Cyagen Biosciences Inc (Suzhou, China). The gRNA target sequences of GPR81^−/−^ mice were as follows: gRNA‐A1: GTCTGTAGCATGTATAGCGTCGG, gRNA‐A2: TAACGATCTCAATAACTCGTCGG, gRNA‐B1: ACCCAGAAATGTACACTCGTGGG, gRNA‐B2: GTGTCGGGTTAGGAACTACCTGG. The gRNA target sequences of UCP1^−/−^ mice were as follows: gRNA‐A1: GGGTACCTTGTTTAGGTGTTTGG, gRNA‐A2: GTGACTTCGAGTCGCTGTATGGG, gRNA‐B1: AACACCTAAACAAGGTACCCAGG, gRNA‐B2: AGTGACTTCGAGTCGCTGTATGG. All mice aged 6–8 weeks were housed in a specific pathogen‐free environment under a 12 h light/dark cycle at a constant temperature of 22 ± 2°C and 50 ± 5% relative humidity. All mice had free access to standard chow and sterile drinking water. All animal experimental procedures were approved by the Institutional Animal Care and Use Committee of the Second Affiliated Hospital of Soochow University (JC‐2024‐09‐015).

### 4.4 Isolation of Primary Preadipocytes

To isolate SVF from mouse WAT, two‐week‐old WT mice and GPR81^−/−^ mice were euthanized via cervical dislocation and immersed in 70% ethanol for 5–10 min. Inguinal WAT was harvested and rinsed in phosphate‐buffered saline (PBS, Biosharp, Beijing, China, Cat number: BL302A). WAT was minced and digested in HEPES‐Collagenase II (Thermo Fisher Scientific, Shanghai, China, Cat number: 17101015) solution at 37°C for 30 min. Digestion was terminated by adding DMEM/F12 (Gibco, Shanghai, China, Cat number: C11330500BT) supplemented with 10% fetal bovine serum (FBS, Biosharp, Beijing, China, Cat number: BL205A) and 1% penicillin‐streptomycin (NCM Biotech, Suzhou, China, Cat number: C100C5) at a 1:1 volume ratio of digestion solution to culture medium. The mixture was centrifuged at 2000 rpm for 10 min at 4°C. The supernatant was discarded, and the cell pellet was resuspended in fresh complete medium. The cell suspension was filtered through a 40 µm cell strainer, then subjected to a second round of centrifugation (2000 rpm, 10 min, 4°C), and the supernatant was discarded. The cell pellet was resuspended in complete medium and seeded into culture plates at a suitable density. Cells were cultured in a humidified incubator with 5% CO_2_. After 4 h, the medium was aspirated, cells were washed 3–5 times with PBS, and fresh complete medium was added.

### 4.5 Differentiating SVF Cells into Adipocytes

Once SVF cells reached full confluence, differentiation induction medium (DMEM/F12 supplemented with 1 µm dexamethasone (Sigma–Aldrich, USA, Cat number: D4902), 10 µg/mL insulin (Beyotime, Shanghai, China, Cat number: P3376), and 0.5 mM isobutylmethylxanthine (Sigma–Aldrich, USA, Cat number: I7018)) was applied for 2 days. The culture medium was then changed to maintenance DMEM (Gibco, Shanghai, China, Cat number: C11995500BT) supplemented with 10 µg/mL insulin and 10% FBS for an additional 2 days. For the final 2 days of differentiation, cells were cultured in DMEM supplemented with 10% FBS only.

### 4.6 siRNA Transfection

Target‐specific siRNAs were designed and synthesized by Thermo Fisher Scientific (Shanghai, China). siRNA transfection into SVF‐derived adipocytes was performed using Lipofectamine RNAiMAX (Thermo Fisher Scientific, Shanghai, China, Cat number: 13778030) according to the manufacturer's instructions. The siRNA target sequences were as follows: siRNA‐MCT1 (Forward primer 5′‐CCAAAUCCAUCACUGUCUUTT‐3′, Reverse primer 5′‐AAGACAGUGAUGGAUUUGGTT‐3′), siRNA‐MCT4 (Forward primer 5′‐GGUCUUUGUGGUGAGCUAUTT‐3′, Reverse primer 5′‐AUAGCUCACCACAAAGACCTT‐3′), siRNA‐GPR4 (Forward primer 5′‐GCAACAUCUAUAUCAGCAUTT‐3′, Reverse primer 5′‐AUGCUGAUAUAGAUGUUGCTT‐3′), siRNA‐GPR81 (Forward primer 5′‐CCUGGAAGUCAAGCACUAUTT‐3′, Reverse primer 5′‐AUAGUGCUUGACUUCCAGGTT‐3′), siRNA‐GPR132 (Forward primer 5′‐GGCUACCACUACCUGCGUUTT‐3′, Reverse primer 5′‐AACGCAGGUAGUGGUAGCCTT‐3′).

### 4.7 Establishment of Different Mouse Sepsis Models

All four sepsis models were established in 6‐8‐week‐old WT C57BL/6 male mice (*n* = 8 per group) for model comparison. Group sizes for animal experiments were determined based on our prior work and were sufficient to detect the expected differences in the primary metabolic endpoints while minimizing animal use in accordance with the 3R principle [24]. For the LPS‐induced sepsis model, mice were intraperitoneally injected with LPS (Sigma‐Aldrich, USA, CAS number: 93572‐42‐0) at a dose of 5 mg/kg. For the CLP‐induced sepsis model, mice were anesthetized via intraperitoneal injection of sodium pentobarbital (50 mg/kg), followed by abdominal skin shaving and thorough disinfection. A midline laparotomy was performed to expose the cecum. Approximately half of the cecum was ligated with a 4‐0 silk suture, preserving intestinal continuity, and the ligated segment was punctured once with a 21‐gauge needle. A small amount of fecal content was gently extruded to ensure patency of the puncture site, after which the cecum was repositioned into the peritoneal cavity. The abdominal incision was closed with continuous silk sutures combined with topical tissue adhesive, and all mice received a subcutaneous injection of 1 mL sterile saline for postoperative fluid resuscitation. For the intraperitoneal *S. aureus*‐induced sepsis models, mice were intraperitoneally injected with 200 µL of a 1 × 10^8^ CFU *S. aureus* (ACTT 43300, USA) suspension. For the intranasal *S. aureus*‐induced sepsis models, anesthetized mice were slowly administered 30 µL of a 6 × 10^8^ CFU *S. aureus* suspension intranasally.

### 4.8 In Vivo and In Vitro Experiments

First, to investigate the effect of UCP1‐mediated pathological WAT browning on sepsis‐induced metabolic disorders, WT and UCP1^−/−^ male mice were randomly divided into 4 groups (*n* = 6 per group): WT Control, WT + LPS, UCP1^−/−^ Control, and UCP1^−/−^ + LPS. Mice in the WT + LPS and UCP1^−/−^ + LPS groups were i.p. injected with LPS (5 mg/kg) to establish a sepsis model. Mice in the WT Control and UCP1^−/−^ Control groups were i.p. injected with an equal volume of saline.

Second, to establish lactate as the upstream causal driver of sepsis‐induced pathological WAT browning and subsequent metabolic disorders, WT male mice were randomly divided into 4 groups (*n* = 8 per group): Control, LPS, Lactate, and LPS + Lactate. Control mice received an i.p saline injection; the LPS group was given 5 mg/kg LPS, the Lactate group was given lactate (0.5 g/kg, Sigma‐Aldrich, USA, Cat number: 71718); the LPS + Lactate group was co‐administered LPS (5 mg/kg) and lactate (0.5 g/kg). A separate cohort of WT male mice was randomly divided into 4 groups (*n* = 8 per group): Control, LPS, LPS + Oxamate, and LPS + 2‐DG. Control and LPS groups were treated as detailed above. For LPS + Oxamate and LPS + 2‐DG groups, mice received i.p. pre‐treatment with oxamate (0.75 g/kg/d, MedChemExpress, Shanghai, China, Cat number: HY‐W013032A) or 2‐DG (0.5 g/kg/d, MedChemExpress, Shanghai, China, Cat number: HY‐13966) 3h before LPS administration. In vitro experiments, WT SVF‐derived adipocytes were firstly incubated with a gradient of lactate concentrations (0, 1.0, 5.0, 10.0, 20.0, 40.0 mM) for 24 h (*n* = 3 biologically independent samples in each group) to verified whether lactate could induce SVF‐differentiated adipocytes browning. Then, to identify the optimal LPS concentration for subsequent cellular experiments, WT SVF‐derived adipocytes were treated with a gradient of LPS concentrations (0, 0.1, 0.2, 0.5, 1.0, 2.0 µg/mL) for 24 h (*n* = 3 biologically independent samples in each group). After LPS dose optimization, adipocytes were pre‐treated with oxamate (20 mM) or 2‐DG (10 mM) for 1 h prior to stimulation with 1 µg/mL LPS (*n* = 3 biologically independent samples in each group).

Third, to identify the specific cell‐surface transporter or receptor mediating lactate‐mediated WAT browning, adipocytes were separately transfected with siRNAs targeting MCT1, MCT4, GPR4, GPR81, and GPR132. After siRNA transfection, adipocytes were treated with LPS stimulation (1 µg/mL) for 24 h (*n* = 3 biologically independent samples in each group). We next investigated the functional role of GPR81 on WAT browning and metabolic disorders. WT and GPR81^−/−^ male mice were randomly divided into 4 groups (*n* = 6 per group): WT Control, WT + LPS, GPR81^−/−^ Control, and GPR81^−/−^ + LPS. Mice in the WT + LPS and GPR81^−/−^ + LPS groups received i.p. LPS (5 mg/kg). Mice in the WT control and GPR81^−/−^ control groups were injected with an equal volume of saline. Meanwhile, WT SVF‐derived adipocytes and GPR81^−/−^ SVF‐derived adipocytes were stimulated with 1 µg/mL LPS (*n* = 3 biologically independent samples in each group). Then, a separate cohort of WT male mice was randomly divided into 4 groups (*n* = 8 per group): Control, LPS, AAV9‐miR‐GPR81, and AAV9‐miR‐GPR81 + LPS. Mice in the AAV9‐miR‐GPR81 and AAV9‐miR‐GPR81 + LPS groups received a local injection of AAV9‐ADP‐eGFP‐miRNA‐GPR81 (short: AAV9‐miR‐GPR81; targeting sequence: AAGATGACCAAAGTCCAGAGG, viral titer: 2.27 × 10^13^ vg/mL) into bilateral inguinal WAT (50 µL per side, 100 µL per mouse), whereas mice in the Control and LPS groups received an equivalent local injection of AAV9‐ADP‐eGFP‐miRNA‐NC (scramble sequence: GTTCTCCGAACGTGTCACGT, viral titer: 2.12 × 10^13^ vg/mL). Both AAV9‐ADP‐eGFP‐miRNA‐GPR81 and AAV9‐ADP‐eGFP‐miRNA‐NC were designed and synthesized by GenePharma (Shanghai, China). Three weeks after viral injection to achieve stable adipose‐specific GPR81 knockdown, mice in the LPS and AAV9‐miR‐GPR81 + LPS groups were intraperitoneally injected with LPS (5 mg/kg) to establish the sepsis models, whereas mice in the Control and AAV9‐miR‐GPR81 groups received an equal volume of saline intraperitoneally.

Finally, to systematically validate whether CTRP6 and p38 served as key downstream mediators of GPR81 signaling responsible for sepsis‐induced WAT browning and metabolic disorders, GPR81^−/−^ male mice were randomly divided into 4 groups (*n* = 6 per group): Control, Vector, AAV9‐CTRP6, and AAV9‐MKK6. Viral vectors were delivered via local injection into bilateral inguinal WAT; each side received 50 µL of virus suspension, yielding a total injection volume of 100 µL per mouse. Mice in the AAV9‐CTRP6 and AAV9‐MKK6 groups received the matching AAV constructs. AAV9‐ADP‐eGFP‐CTRP6 (vector ID: P3407‐1, viral titer: 1.05 × 10^13^ vg/mL) and AAV9‐ADP‐eGFP‐MKK6 (constitutively active MKK6, vector ID: P3326‐1, viral titer: 2.60 × 10^13^ vg/mL) were designed and synthesized by GenePharma (Shanghai, China). Mice in the Control and Vector groups received an equal volume of saline and empty viral vector via local injection into bilateral inguinal WAT, respectively. All mice were housed for 4 weeks following viral delivery, and metabolic measurements were conducted on the last day of this housing period. A separate cohort of WT male mice was randomly divided into 4 groups (*n* = 8 per group): Control, LPS, LPS + Rosiglitazone, and LPS + SB203580. Mice in the control and LPS groups received the same treatments as described above. For the LPS + Rosiglitazone and LPS + SB203580 groups, mice received i.p. pre‐treatment with rosiglitazone (15 mg/kg/d, MedChemExpress, Shanghai, China, Cat number: HY‐17386), or SB203580 (20 mg/kg/d, MedChemExpress, Shanghai, China, Cat number: HY‐10256) 3 h before LPS administration. For cellular validation, WT SVF‐derived adipocytes were treated with rosiglitazone (10 µm) or SB203580 (10 µm) for 1 h before LPS stimulation (1 µg/mL) (*n* = 3 biologically independent samples in each group).

72 h after in vivo sepsis induction, mice were subjected to humane euthanasia prior to rapid isolation of serum, inguinal WAT, and EDL for further assays. Cellular phenotypes and molecular markers were assessed 24 h after LPS treatment in all in vitro experiments.

### 4.9 Western Blot

Total extracted protein was separated by 12.5% sodium dodecyl sulfate polyacrylamide gel electrophoresis, then electrotransferred onto polyvinylidene difluoride membranes at a constant current of 300 mA. After blocking, membranes were incubated with the primary antibodies against target proteins overnight at 4°C, including GPR81 (Novus Biologicals, USA, Cat number: NBP1‐51956, 1:1000), CTRP6 (Abcam, UK, Cat number: AB36900, 1:1000), p‐p38 (Cell Signaling Technology, CST, USA, Cat number: 4511, 1:1000), p38 (CST, USA, Cat number: 8690, 1:1000), UCP1 (Proteintech, Wuhan, China, Cat number: 83870, 1:1000), MURF‐1 (Abcam, UK, Cat number: AB172479, 1:1000), MAFBx (Abcam, UK, Cat number: AB168372, 1:1000), and β‐tubulin (Proteintech, Wuhan, China, Cat number: 10094, 1:5000). Membranes were washed three times with tris‐buffered saline with Tween‐20 and incubated with corresponding secondary antibodies at room temperature for 1 h. Protein bands were visualized using an enhanced chemiluminescence substrate, and band intensities were quantified using ImageJ software.

### 4.10 Grip Strength

Forelimb grip strength was measured using a Chatillon DFX II grip strength meter (USA). Mice were allowed to firmly grasp the metal bar with forepaws, and their tails were pulled backward at a consistent speed until release. Each mouse was tested five times, and the mean value was recorded as the final grip strength.

### 4.11 Serum, WAT and Adipocyte Lactate Measurement

Lactate concentrations in serum, WAT, and adipocytes were measured using a lactate assay kit (Abbkine, Wuhan, China, Cat number: KTB1100) according to the manufacturer's instructions.

### 4.12 Inflammatory Cytokines Measurement

Levels of IL‐1β, IL‐6, and TNF‐α in serum and WAT were measured using ELISA kits (R&D Systems, Minneapolis, MN, USA) according to t the manufacturer's instructions.

### 4.13 Fasting Blood Glucose, Fasting Serum Insulin and Insulin Resistance Measurements

Fasting blood glucose was measured via a blood glucose meter (Roche), and fasting serum insulin was measured via an insulin ELISA kit (RayBiotech, USA, Cat number: ELM‐Insulin‐1). The values of HOMA‐IR were calculated as follows:

$$\begin{array}{cc} & \mathrm{HOMA}\;-\;\mathrm{IR}=\\ & \frac{\mathrm{fasting}\;\mathrm{blood}\;\mathrm{glucose}\;(\mathrm{mmol}/L)\;\times \;\mathrm{fasting}\;\mathrm{serum}\;\mathrm{insulin}\;(\mathrm{mIU}/\mathrm{ml})}{22.5} \end{array}$$

### 4.14 Metabolic Testing

On day 3 after sepsis modeling, each mouse was individually housed in a metabolic cage (Columbus Instruments, CLAMS, USA) under a 12 h light/dark cycle. Daily food intake, body weight, and EE were continuously recorded. Total daily EE is reported in absolute units (kcal/day). Because body weight differed across experimental groups and is a major determinant of EE, between‐group comparisons of EE were additionally performed by analysis of covariance (ANCOVA) with body weight as the covariate, alongside the unadjusted data.

### 4.15 H&E Staining

WAT and EDL were fixed in 10% formalin and embedded in paraffin. Serial 5 µm‐thick sections were sliced with a microtome and mounted on glass slides. Following deparaffinization and rehydration, the sections were stained with H&E. Stained sections were observed under an ordinary light microscope, and the cell diameter was quantified using ImageJ software. All quantification was performed by an operator blinded to group allocation, with sections coded and re‐randomized prior to analysis.

### 4.16 Immunohistochemistry

Following deparaffinization and rehydration, paraffin‐embedded WAT sections underwent antigen retrieval in citrate buffer for 15 min. Sections were then incubated with 3% hydrogen peroxide for 10 min to block endogenous peroxidase activity, and incubated overnight with the primary antibody against UCP1 (1:400) at 4°C. After PBS washing, sections were incubated with the secondary antibody at room temperature for 1 h, and images were captured under an ordinary light microscope. Quantification of UCP1 immunostaining was performed in a blinded manner as described above.

### 4.17 Immunofluorescence

Cultured adipocytes were gently washed twice with PBS, followed by fixation, permeabilization, and non‐specific binding blocking. Cells were incubated with the primary antibody against UCP1 (1:400) overnight at 4°C. After three washes with PBS containing 0.05% Tween‐20, cells were incubated with fluorescein‐conjugated secondary antibody in the dark for 1 h. Cell nuclei were stained with 4',6‐diamidino‐2‐phenylindole (DAPI), and slides were sealed with anti‐fade mounting medium. Fluorescent images were acquired under a fluorescence microscope. Quantification of fluorescence signals was performed in a blinded manner as described above.

### 4.18 Targeted Metabolomics

Twenty human omental WAT samples (10 from septic patients, 10 from non‐septic patients) stored at −80°C were analyzed via UHPLC‐MRM‐MS/MS for targeted quantification of 146 energy metabolism‐related metabolites. Briefly, 25 mg frozen adipose tissue with grinding beads was mixed with 500 µL methanol‐water extraction buffer containing isotope internal standards, vortexed for 30 s, ground for 4 min, and ice‐bath‐sonicated for 5 min. After standing at −40°C for 1 h, samples were centrifuged at 12,000 rpm for 15 min at 4°C, and supernatants were collected for subsequent instrumental analysis. Mixed calibration standards containing identical isotopic internal standards were serially diluted from individual 10 mmol/L metabolite stock solutions. Two chromatographic separation modes were performed on an Agilent 1290 Infinity II UHPLC system: reversed‐phase separation using an ACQUITY UPLC BEH C18 column, and hydrophilic interaction liquid chromatography (HILIC) separation using an Atlantis Premier BEH Z‐HILIC column. The sample tray temperature was maintained at 6 °C, and the injection volume was fixed at 2 µL for all samples. Metabolite detection was performed on a Sciex Triple Quad 6500+ mass spectrometer (MS) equipped with an IonDrive Turbo V ESI ion source operated in both positive and negative multiple reaction monitoring mode. Raw MS data were acquired using SCIEX Analyst Work Station Software v1.7.3, and metabolite quantification was processed via BIOTREE BioBud v2.0.4 software. Calibration curves were generated based on peak area ratios of target analytes to corresponding internal standards. Calibration points with calculated accuracy outside the 80%‐120% range were removed as outliers. The lower limit of detection (S/N = 3) and lower limit of quantification (S/N = 10) of each metabolite were determined in accordance with US FDA analytical guidelines. Tissue metabolite concentration (C_M_, nmol/g) was calculated as follows:

$$C_{M}=\frac{Calculated\;Concentration\;\left(nmol/L\right)\times Extraction\;Volume\;\left(\mu L\right)}{Sample\;weight\;(mg)\times Concentration\;factor\times 1000}$$

### 4.19 Statistical Analysis

The collection of experimental data followed a randomized approach. Briefly, the ‘RAND’ function in Microsoft Excel was used to generate a random value for each mouse, and mice were subsequently sorted and allocated to experimental groups based on these random values. All data are presented as the mean ± standard error of the mean (SEM). All statistical analyses were performed using GraphPad Prism (Version 10.4.2, GraphPad Software, Boston, Massachusetts, USA). The Shapiro‐Wilk test was used to assess normality, and Levene's test was used to assess homogeneity of variance. For normally distributed data with equal variances, two‐group comparisons were performed using the unpaired two‐tailed Student's t‐test, and comparisons among three or more groups were performed using one‐way or two‐way ANOVA followed by Tukey's post‐hoc test; normally distributed data with unequal variances were analyzed using Welch's t‐test. For non‐normally distributed data, two‐group comparisons were performed using the Mann‐Whitney U test; comparisons among three or more groups were performed using the Kruskal‐Wallis test followed by Dunn's multiple comparison test. All pairwise P values from multi‐group comparisons are adjusted for multiple testing. Categorical variables were compared using Fisher's exact test owing to the small sample size. Linear associations were evaluated using Pearson correlation analysis and simple linear regression. Survival was compared using the Kaplan‐Meier method with the log‐rank test. A *P* value < 0.05 was considered statistically significant.

## Author Contributions

**Chunliang Zheng**: investigation, data curation, validation, writing – review and editing, writing – original draft. **Xiaohua Li**: data curation, investigation, validation, writing – original draft, writing – review and editing. **Shuo Li**: investigation, validation, writing – original draft, writing – review and editing, data curation. **Chungen Xing**: conceptualization, methodology, investigation, writing – original draft, writing – review and editing. **Chun Cao**: conceptualization, methodology, writing – original draft, writing – review and editing, funding acquisition, investigation, data curation.

## Funding

This study was supported by the National Natural Science Foundation of China (82202369), Jiangsu Key Laboratory for Organoid Creation and Precision Medicine (214267), Suzhou Gusu Health Talents Research Project (GSWS2025056 and GSWS2025188), and the Postgraduate Research&Practice Innovation Program of Jiangsu Province (26CXJH5991).

## Conflicts of Interest

The authors declare no conflicts of interest.

## Supporting information




**Supporting File 1**: advs78487‐sup‐0001‐Figures.zip.

**Supporting File 2**: advs78487‐sup‐0002‐TableS1.docx.

**Supporting File 3**: advs78487‐sup‐0003‐TableS2.docx

## Data Availability

The data that support the findings of this study are available on reasonable request from the corresponding author.
