# Supplementary material for: Lactate Drives Pathological Browning of White Adipose Tissue to Aggravate Sepsis‐Associated Metabolic Disorders in a GPR81‐Dependent Manner
Source: Adv Sci (Weinh). 2026 Oct 29:e78487. Online ahead of print. doi: 10.1002/advs.78487 (PMC13720389; doi:10.1002/advs.78487)
Supplement: Supplementary file 1 [file ADVS-9999-e78487-s001.docx]

**Table S2 Coefficient of variation analysis of metabolic and functional phenotypes to assess intra-group stability across multiple mouse sepsis models.**

| **Variable** | **Control** | | **LPS** | | **CLP** | | **i.p. *S. aureus*** | | **i.n. *S. aureus*** | |
| --- | --- | --- | --- | --- | --- | --- | --- | --- | --- | --- |
|  | **mean ± SEM** | **CV (%)** | **mean ± SEM** | **CV (%)** | **mean ± SEM** | **CV (%)** | **mean ± SEM** | **CV (%)** | **mean ± SEM** | **CV (%)** |
| Lactate in WAT  (mmol/g) | 0.0705 **±** 0.0251 | 35.60 | 0.2512 **±** 0.0694 | 27.64 | 0.3312 **±** 0.1198 | 36.18 | 0.2637 **±** 0.0923 | 34.98 | 0.2249 **±** 0.0623 | 27.70 |
| Serum lactate  (mmol/L) | 1.1290 **±** 0.3471 | 30.75 | 2.6710 **±** 0.6952 | 26.03 | 2.9810 **±** 1.328 | 44.53 | 2.5640 **±** 0.8837 | 34.47 | 2.3770 **±** 0.7488 | 31.51 |
| Total daily EE  (kcal/d) | 11.90 **±** 1.228 | 10.33 | 15.69 **±** 1.344 | 8.566 | 17.03 **±** 2.662 | 15.64 | 16.33 **±** 2.958 | 18.11 | 14.54 **±** 2.314 | 15.91 |
| Grip strength  (gF) | 338.6 **±** 80.83 | 23.87 | 190.9 **±** 61.12 | 32.02 | 172.9 **±** 88.80 | 51.37 | 184.5 **±** 86.51 | 46.89 | 211.8 **±** 74.95 | 35.38 |
| HOMA-IR | 0.0166 **±** 0.0072 | 43.23 | 0.0725 **±** 0.0157 | 21.63 | 0.0827 **±** 0.0343 | 41.44 | 0.0690 **±** 0.0259 | 37.49 | 0.0576 **±** 0.0148 | 25.73 |

Abbreviations: LPS, lipopolysaccharide; CLP, cecal ligation and puncture; i.p., intraperitoneal; i.n., intranasal; *S. aureus, Staphylococcus aureus*; SEM, standard error of the mean; CV, coefficient of variation; WAT, white adipose tissue; EE, energy expenditure; HOMA-IR, homeostasis model assessment of insulin resistance.
