# Supplementary material for: Lactate Drives Pathological Browning of White Adipose Tissue to Aggravate Sepsis‐Associated Metabolic Disorders in a GPR81‐Dependent Manner
Source: Adv Sci (Weinh). 2026 Oct 29:e78487. Online ahead of print. doi: 10.1002/advs.78487 (PMC13720389; doi:10.1002/advs.78487)
Supplement: Supplementary file 2 [file ADVS-9999-e78487-s002.docx]

**Table S1 Comparison of demographic, clinical and laboratory characteristics between non-septic and septic patients.**

| **Variable** | **Non-sepsis (n=10)** | **Sepsis (n=10)** | **P value** |
| --- | --- | --- | --- |
| Sex |  |  |  |
| Male | 5 (50%) | 9 (90%) | 0.141 |
| Female | 5 (50%) | 1 (10%) |  |
| Age (years) | 60 (30, 71) | 65 (57, 69) | 0.245 |
| BMI (kg/m^2^) | 24.14 (22.60, 26.31) | 24.49 (22.05, 26.11) | 0.983 |
| SOFA | 2.00 (0.75, 3.00) | 5.00 (4.00, 7.00) | <0.001*** |
| APACHE II | 5.50 (3.00, 7.25) | 12.50 (10.75, 16.50) | <0.001*** |
| Preoperative laboratory examination |  |  |  |
| WBC (× 10^9^/L) | 4.8 (4.2, 5.7) | 17.5 (14.9, 23.9) | <0.001*** |
| Neutrophil (× 10^9^/L) | 2.9 (2.4, 3.8) | 11.7 (4.0, 18.3) | 0.001** |
| Lymphocyte (× 10^9^/L) | 1.2 (1.0, 1.8) | 1.0 (0.5, 1.1) | 0.051 |
| Eosinophil (× 10^9^/L) | 0.10 (0.05, 0.18) | 0.01 (0, 0.07) | 0.006** |
| Basophil (× 10^9^/L) | 0.02 (0.01, 0.03) | 0.03 (0.01, 0.03) | 0.872 |
| Platelet (× 10^9^/L) | 213 (162, 236) | 112 (80, 283) | 0.138 |
| CRP (mg/L) | 9.2 (6.1, 21.5) | 61.8 (43.4, 108.8) | 0.002** |
| PCT (ng/mL) | 0.34 (0.18, 0.49) | 5.94 (1.18, 39.82) | <0.001*** |
| Hb (g/L) | 139 (128, 144) | 125 (116, 140) | 0.062 |
| Albumin (g/L) | 42.8 (40.5, 43.9) | 35.8 (30.9, 39.6) | 0.003** |
| Globulin (g/L) | 24.4 (21.2, 28.5) | 23.4 (21.9, 27.0) | 0.828 |
| Prealbumin (g/L) | 0.22 (0.16, 0.23) | 0.18 (0.09, 0.27) | 0.404 |
| INR | 1.03 (0.99, 1.09) | 1.06 (0.99, 1.28) | 0.206 |
| APTT (s) | 34.3 (32.8, 35.3) | 37.7 (31.4, 41.8) | 0.392 |
| PT (s) | 13.5 (12.9, 14.1) | 13.9 (13.3, 16.1) | 0.087 |
| TT (s) | 17.3 (16.6, 17.6) | 16.7 (16.3, 17.6) | 0.433 |
| Fibrinogen (mg/dL) | 3.35 (3.02, 4.10) | 4.11 (2.91, 4.64) | 0.498 |
| TBil (μmol/L) | 33.4 (11.6, 94.0) | 46.3 (29.5, 120.0) | 0.190 |
| ALT (U/L) | 95 (30, 181) | 311 (25, 469) | 0.036* |
| AST (U/L) | 54 (25, 200) | 198 (16, 315) | 0.271 |
| ALP (U/L) | 138 (92, 204) | 135 (82, 243) | 0.775 |
| LDH (U/L) | 232 (145, 281) | 208 (149, 324) | 0.786 |
| GGT (U/L) | 124 (30, 221) | 385 (72, 474) | 0.049* |
| CK (U/L) | 65 (51, 97) | 97 (55, 306) | 0.147 |
| Cr (μmol/L) | 60 (51, 71) | 142 (51, 196) | 0.013* |
| TC (mmol/L) | 4.43 (3.96, 5.07) | 3.88 (2.72, 4.76) | 0.179 |
| TG (mmol/L) | 1.00 (0.72, 1.42) | 1.07 (0.89, 1.59) | 0.667 |

Abbreviations: BMI, body mass index; WBC, white blood cell; CRP, C-reactive protein; PCT, procalcitonin; Hb, hemoglobin; INR, international normalized ratio; APTT, activated partial thromboplastin time; PT, prothrombin time; TT, thrombin time; TBil, total bilirubin; ALT, alanine aminotransferase; AST, aspartate aminotransferase; ALP, alkaline phosphatase; LDH, lactate dehydrogenase; GGT, gamma glutamyl transferase; CK, creatine kinase; Cr, creatinine; TC, total cholesterol; TG, triglycerides. *p < 0.05, **p < 0.01, ***p < 0.001.
